# Current updates relating to treatment for interstitial cystitis/bladder pain syndrome: systematic review and network meta-analysis

**DOI:** 10.1186/s12894-024-01485-w

**Published:** 2024-04-24

**Authors:** Jae Joon Park, Kwang Taek Kim, Eun Ji Lee, Joey Chun, Serin Lee, Sung Ryul Shim, Jae Heon Kim

**Affiliations:** 1 Department of Urology, Soonchunhyang University Seoul Hospital, Soonchunhyang University College of Medicine, 59 Daesagwan-ro, Yongsangu, Seoul, 04401 Republic of Korea; 2 Department of Urology, Gil Medical Center, Gachon University College of Medicine, Incheon, Republic of Korea; 3 Department of Radiology, Soonchunhyang University Seoul Hospital, Soonchunhyang University College of Medicine, Seoul, Republic of Korea; 4 Cranbrook Kingswood Upper School, Bloomfield Hills, Michigan United States; 5 Department of Biochemistry, Case Western Reserve University, Cleveland, Ohio United States; 6 Department of Biomedical Informatics, College of Medicine, Konyang University, 158 Gwanjeodong-ro, Seo-gu, Daejeon, 35365 Republic of Korea; 7 Konyang Medical data Research group-KYMERA, Konyang University Hospital, Daejeon, Republic of Korea

**Keywords:** Cystitis, Interstitial, Pain, Lower Urinary Tract Symptoms, Urinary Bladder, Overactive, Medication Therapy Management, Administration, Intravesical, Physical Therapy Modalities

## Abstract

**Background:**

Despite the publication of several meta-analyses regarding the efficacy of certain therapies in helping individuals with interstitial cystitis (IC) / bladder pain syndrome (BPS), these have not provided a comprehensive review of therapeutic strategies. The study aimed to determine the efficacy of various therapies for IC/BPS and identify potential moderating factors using randomized controlled trials (RCTs).

**Methods:**

We queried the PubMed, Cochrane, and Embase databases to identify prospective RCTs using inclusion criteria: 1) patients diagnosed with IC, 2) interventions included relevant treatments, 3) comparisons were a specified control or placebo, 4) outcomes were mean differences for individual symptoms and structured questionnaires. The pairwise meta-analysis and network meta-analysis (NMA) were performed to compare the treatments used in IC/BPS. Hedges’ *g* standardized mean differences (SMDs) were used for improvement in all outcomes using random-effects models. Efficacy outcomes included individual symptoms such as pain, frequency, urgency, and nocturia, as well as structured questionnaires measuring IC/BPS symptoms.

**Results:**

A comprehensive literature search was conducted which identified 70 RCTs with 3,651 patients. The analysis revealed that certain treatments, such as instillation and intravesical injection, showed statistically significant improvements in pain and urgency compared to control or placebo groups in traditional pairwise meta-analysis. However, no specific treatment demonstrated significant improvement in all outcomes measured in the NMA. The results of moderator analyses to explore influential variables indicated that increasing age was associated with increased nocturia, while longer follow-up periods were associated with decreased frequency.

**Conclusion:**

This systematic review and meta-analysis provide insights into the efficacy of various treatments for IC. Current research suggests that a combination of therapies may have a positive clinical outcome for patients with IC, despite the fact that treatment for this condition is not straightforward.

**Trial registration:**

PROSPERO CRD42022384024

**Supplementary Information:**

The online version contains supplementary material available at 10.1186/s12894-024-01485-w.

## Background

A constellation of incapacitating systems, which encompass discomfort in the suprapubic region, nocturia and micturition frequency and urgency, are characteristic of interstitial cystitis (IC), a condition also referred to as bladder pain syndrome (BPS). Typically, such symptoms occur when the bladder is full, and dissipate once urine has been passed [1, 2]. In addition to these symptoms, a major complaint of patients suffering from IC is pelvic pain, which originates from the urethra, vagina or rectum, amongst other viscera [3]. There is at present no standard definition for this condition, nor consensus regarding its management, recommended therapy or the treatment period required [4].

A range of drugs directed towards the treatment of IC have been the topic of recent debate, although the largest percentage of patients cured of the condition remains in the region of 60% [5]. It has been demonstrated that numerous factors are involved in the pathogenesis of IC. These include autoimmune phenomena [6], injury to the urothelial glycosaminoglycan layer [7], neurological discomfort and inflammatory processes [8].

Despite the publication of several meta-analyses as well as a network meta-analysis (NMA) [9, 10], these have not provided a comprehensive review of therapeutic strategies. The lack of clarity regarding the efficacy of certain therapies in helping individuals with IC, owing to the low or very low degrees of supportive evidence, was expressed in a recent Cochrane review [9]. Although the results of numerous studies can be accessed, the majority only included small populations and so the outcomes of therapy could not be accurately evaluated. The same issue applied to a further NMA, and so the results were not apposite for the current work. Thus, studies with higher sample numbers and which are specifically targeted towards this condition are required to enhance the present quality of evidence. Randomized controlled trials (RCTs) have been added by the current authors to the literature, and they have concurrently carried out a traditional pairwise meta-analysis and an NMA to offer more evidence-based conclusions.

There are numerous types of therapy available for IC, some of which are extremely complicated, and so as well as carrying out an NMA, a general meta-analysis by effect variables has also been performed. Additionally, meta-regression analysis was applied to ascertain the moderator effect. The study is underpinned by the hypothesis that a high level of therapeutic success can be achieved in patients with IC using a combination of approaches; this theory could subsequently be tested in RCTs.

## Methods

The systematic review and meta-analysis was conducted in accordance with the Preferred Reporting Items for Systematic Reviews and Meta-Analyses statement [11, 12]. This study completed PROSPERO registration (CRD42022384024).

### Data sources and literature searches

A systematic literature search was conducted in the PubMed, Embase, and Cochrane databases using Medical Subject Headings terms and text keywords through April 2023 (eTable 1 in the Supplement). The subject headings and text keywords included those related to the population of interest (i.e., “IC”, “BPS”), intervention (relevant treatments such as “medication”, “instillation”, “intravesical injection”, “physical therapy” and/or others), comparison (“placebo” or “control”), and outcomes of treatments: 1) individual symptoms (“pain, frequency”, “urgency” and “nocturia”), 2) structured questionnaires (“interstitial cystitis problem index” [ICPI], “interstitial cystitis symptom index” [ICSI], “pelvic pain — urgency” – “frequency symptom scale” [PUF] and “functional bladder volume” [FBV]). Search terms were categorized using Boolean operators (e.g., AND, OR, NOT). Only RCTs were included in this meta-analysis. This search was conducted regardless of language or study type. Two independent researchers (SR Shim and JH Kim) identified additional studies by manually searching trial databases and reference lists.

### Study selection

Study inclusion criteria were as follows: patients diagnosed with IC/BPS, interventions included relevant treatments, comparisons were specified as control or placebo, outcomes were mean differences for individual symptoms (pain, frequency, urgency and nocturia) and structured questionnaires (ICPI, ICSI, PUF and FBV) using an RCT study design. The specific study selection was described in eTable 6 in the Supplement.

### Meta-analysis assessment of outcome findings and statistical analysis

The specific data extraction and calculation methods [13] were listed in eTable 6 in the Supplement. For traditional pairwise meta-analysis, the standardized mean difference (SMD) along with their 95% confidence intervals (Cls) were calculated for continuous variables. The random-effects model calculated by restricted maximum-likelihood estimator (REML) was used to obtain the pooled overall SMD and 95% CIs for outcomes [14].

Each moderator was subjected to a meta-regression analysis for continuous variables (e.g., number of patients, age and follow-up period) and categorical variables (e.g., treatments, country, cystoscopy and Hunner lesion group). To analyse potential moderators, the study estimated the variance of the true effects using a REML estimator.

For Bayesian NMA, specific graphical analysis was completed using the “gemtc” package in R software v.4.2.1 (R Foundation for Statistical Computing) [15]. The Markov chain Monte Carlo (MCMC) simulation method and surface under the cumulative ranking curve (SUCRA) [15, 16] generation used for Bayesian NMA, and the potential publication bias [17] and quality assessment [18] were described in eTable 6 in the Supplement. A two-sided *P*-value of ≤ .05, or not containing a null value (SMD = 0) within the 95% CI was considered to be significant.

## Results

### Study selection

**Fig. 1:**
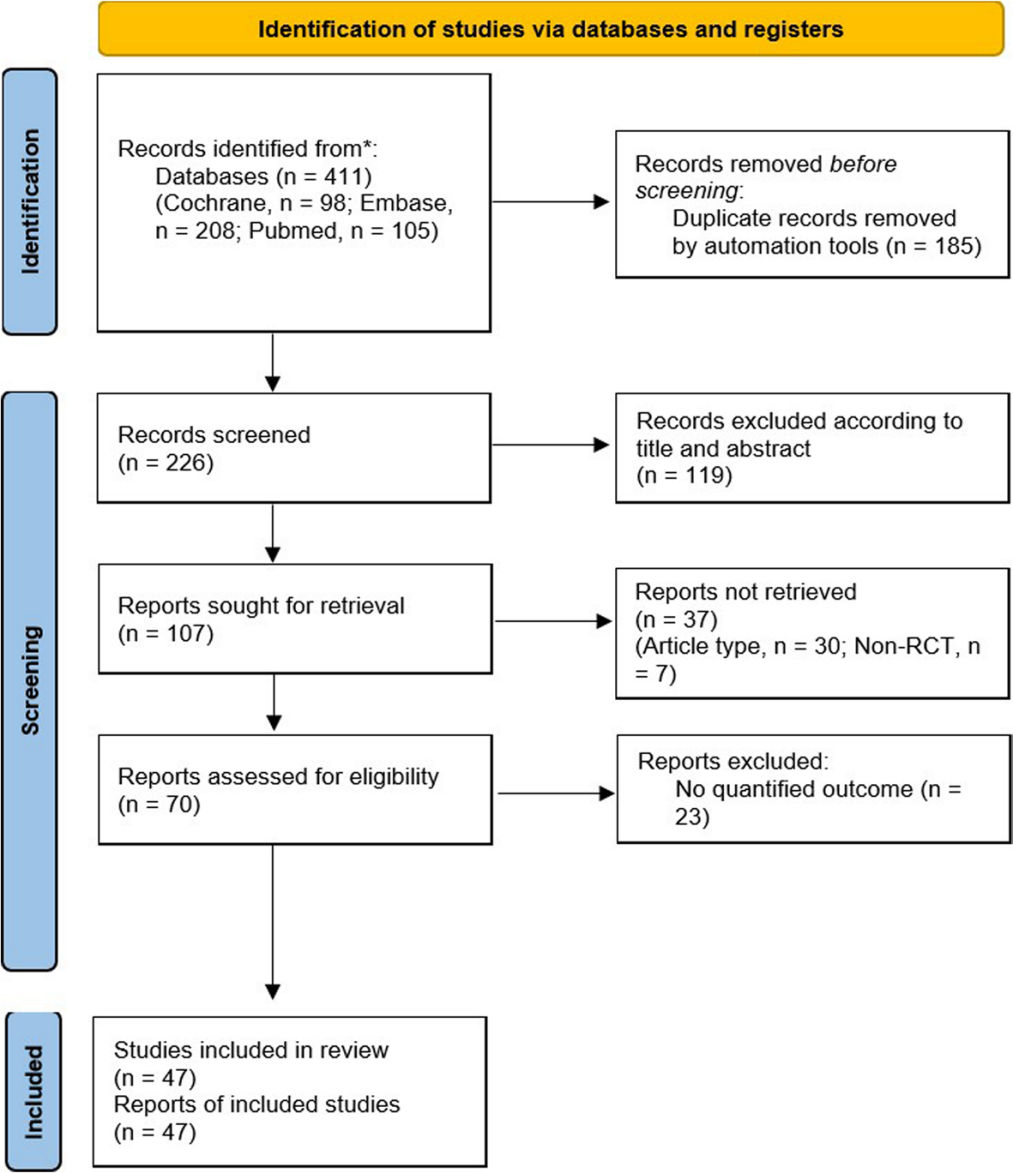
PRISMA flowchart

The initial search identified a total of 411 articles from different electronic databases (PubMed, *n* = 105; Cochrane, *n* = 98; Embase, *n* = 208). Of these, 185 studies contained overlapping data or appeared in more than one database and were therefore excluded. After screening the titles and abstracts, 119 studies were eliminated as they were trial registrations and abstracts only. Among 107 full-text articles, 37 studies were further excluded for the following reasons: not an original article type, *n* = 30 and not an RCT design, *n* = 7. Finally, 70 studies met the selection criteria for qualitative analysis (eTable 2 & 3 in the Supplement) and 47 studies for quantitative synthesis (eTable 4 in the Supplement and Fig. 1).

A systematic review and meta-analysis of the 47 studies involving a total of 3,651 patients were conducted to assess the detailed differences and subject descriptions provided in eTable 4 in the Supplement. All studies were RCTs and conducted in Western, Eastern, and Middle Eastern countries. The mean age range was 30.1 to 65.5 years, and the follow-up period ranged from one to 24 months. The specific treatments and outcomes displayed in eTable 4 in the Supplement include: 1) medication or system injection (i.e., analgesics, antidepressants, new immune modulator, old immune modulator), 2) instillation (i.e., botulinum toxin [BTX], sodium chondroitin sulfate [CS], sodium hyaluronic acid [HA]), 3) intravesical injection (i.e., BTX, RTX, anesthetics), 4) physical therapy (i.e., radiofrequency, nerve stimulation), 5) others (i.e., dietary, hydrogen-rich water, video intervention).

### Outcome findings from pairwise meta-analysis

**Fig. 2:**
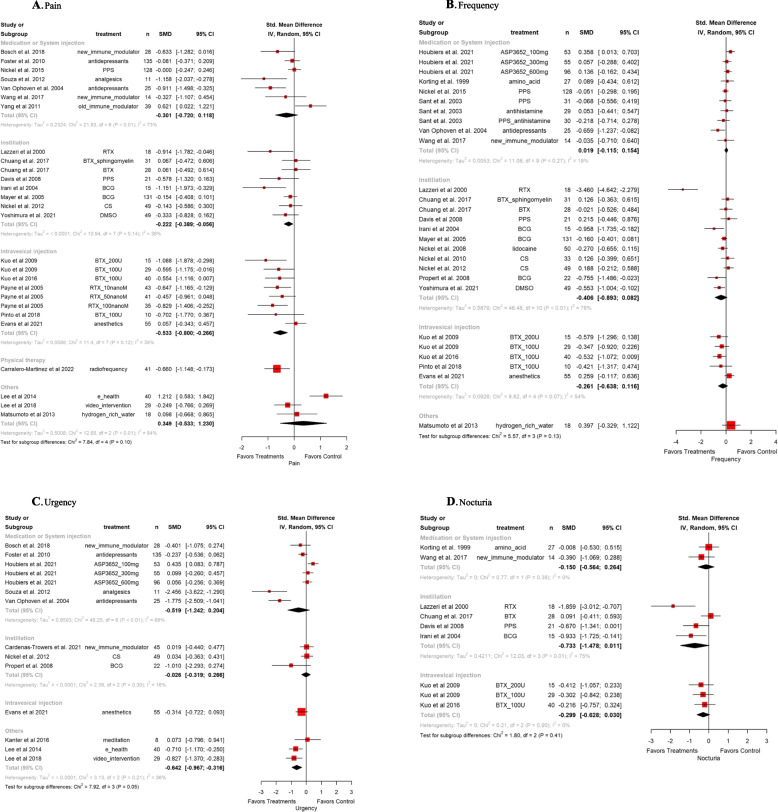
Forest plots for individual symptoms of pain, frequency, urgency, and nocturia (clockwise) in pairwise meta-analysis. SMD, standardized mean difference; CI, confidence interval; PPS, pentosane polysulfate; MPD, monophosphate dehydrogenase; HA, hyaluronic acid; CS, chondroitin sulfate; HL, heparin sodium-lidocaine; BTX, botulinum toxin

**Fig. 3:**
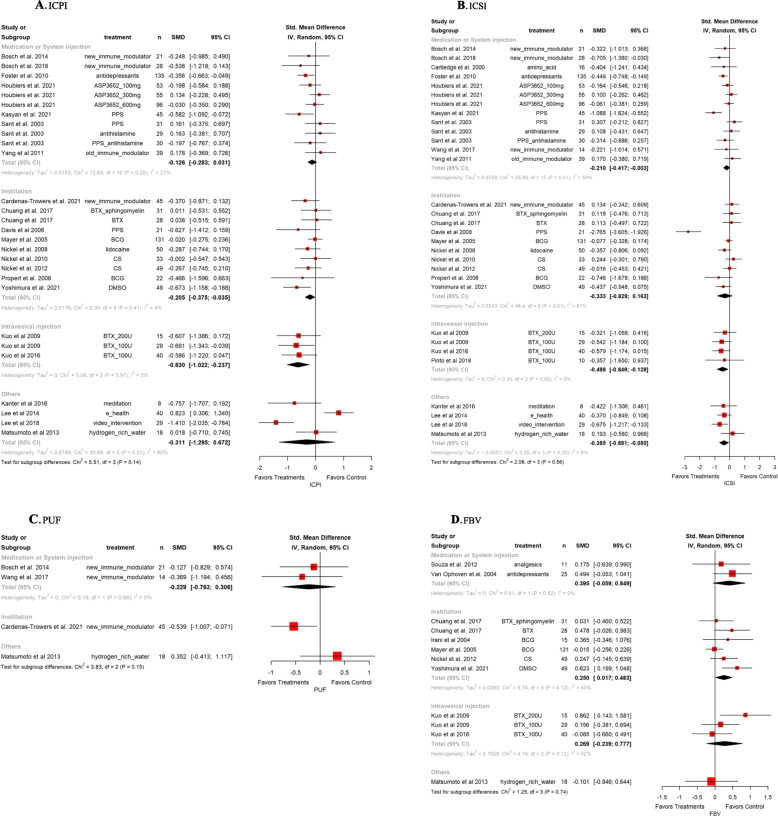
Forest plots for structured questionnaires of ICPI, ICSI, PUF, and FBV (clockwise) in pairwise meta-analysis. SMD, standardized mean difference; CI, confidence interval; PPS, pentosane polysulfate; MPD, monophosphate dehydrogenase; HA, hyaluronic acid; CS, chondroitin sulfate; HL, heparin sodium-lidocaine; BTX, botulinum toxin; ICPI, interstitial cystitis problem index; ICSI, interstitial cystitis symptom index; PUF, pelvic pain – urgency - frequency symptom scale; FBV, functional bladder volume

The pooled SMDs for overall pain assessment between treatments versus the control group was -0.33 (95% CI: -0.52, -0.14). The heterogeneity test produced a Higgins’ I^2^ was 65.5%. In the subgroup analysis by outcome measures, the SMDs were -0.22 (95% CI; -0.39, -0.06) for instillation, and -0.53 (95% CI; -0.80, -0.27) for intravesical injection. The pooled SMDs for overall urgency assessment between treatments versus the control group was -0.40 (95% CI: -0.75, -0.05). The heterogeneity test produced a Higgins’ I^2^ was 79.8%. In the subgroup analysis by outcome measures, the SMD of the others group was statistically significant -0.64 (95% CI; -0.97, -0.32). The pooled SMDs for overall nocturia assessment between treatments versus the control group was -0.37 (95% CI: -0.62, - 0.11). The heterogeneity test produced a Higgins’ I^2^ was 44.5%. In the subgroup analysis by outcome measures, there were no statistically significant groups (Fig. 2). The pooled SMDs for overall ICPI was -0.22 (95% CI: -0.37, -0.07). The heterogeneity test produced a Higgins’ I^2^ was 54.0%. In the subgroup analysis by outcome measures, the SMDs were -0.21 (95% CI; -0.38, -0.04) for instillation, and -0.63 (95% CI; -1.02, -0.28) for intravesical injection. The pooled SMDs for overall ICSI was -0.20 (95% CI: -0.32, -0.08). The heterogeneity test produced a Higgins’ I^2^ was 32.0%. In the subgroup analysis by outcome measures, the SMDs were -0.21 (95% CI; -0.42, -0.01) for medication or system injection, -0.49 (95% CI; -0.85, -0.13) for intravesical injection, and -0.39 (95% CI; -0.69, -0.08) for others. All statistical analysis results were based on clinical practice. (Fig. 3).

### Outcome findings from NMA

**Fig. 4:**
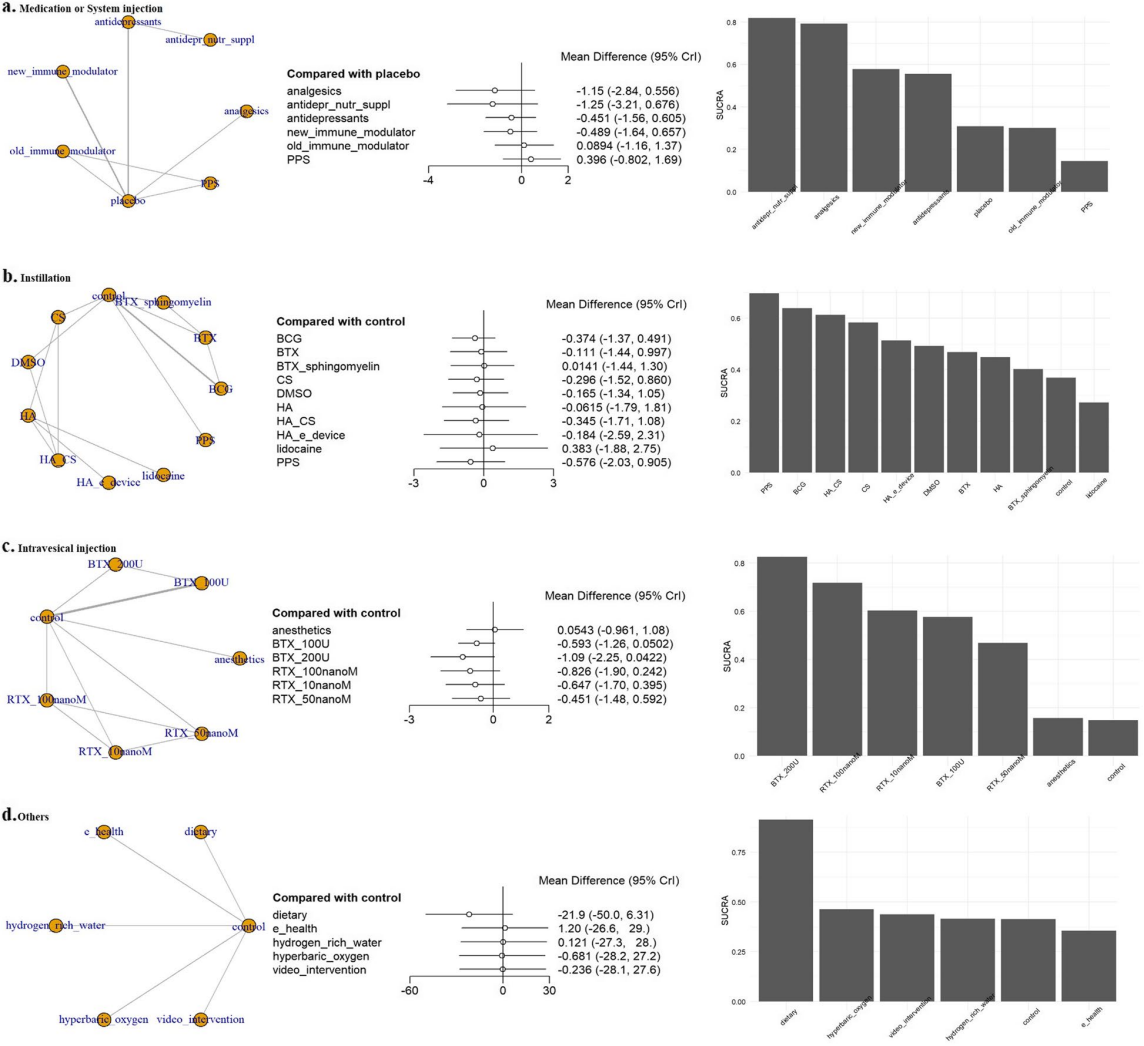
Network plots, forest plots, and SUCRA values of pain in network meta-analysis

Using a Bayesian network meta-analysis to simultaneously examine the differences in each treatment for each outcome measure, the study found that no specific treatment showed statistically significant improvement compared to the control group in all outcomes (pain, frequency, urgency, nocturia, ICPI, ICSI, PUF and FBV) (Fig. 4, and eFigure 1 to 6 in the Supplement). The consistency test showed that the direct and indirect comparison of all outcomes was consistent, so we applied the consistency model in this study (all *P* > 0.05). All statistical analysis results were based on clinical practice.

### Moderator analysis

The study also considered the potential moderating roles of the following variables using meta-regression analysis (Table 1 and eTable 5 in the Supplement). With increasing age, a statistically significant increase was found in nocturia (*β* = 0.063, *P* = 0.005*)*, worsening by 3.6%. With an increasing follow-up period, a statistically significant decrease in frequency (*β* = -0.040, *P* = 0.014*)* was found, improving by approximately 2.3%.

**Table 1:** Effects of moderators for interstitial cystitis in pairwise meta-analysis

	**Pain**						**Frequency**						**Urgency**						**Nocturia**					
Variables	*k*	*β*	SMD	95% CI		*p*	*k*	*β*	SMD	95% CI		*P*	*k*	*β*	SMD	95% CI		*p*	*k*	*β*	SMD	95% CI		*p*
No. of total patients	27	0.004		0.000	0.009	0.08	27	0.003		-0.002	0.008	0.21	14	0.008		-0.002	0.019	0.10	9	0.025		-0.004	0.053	0.09
Age	25	0.010		-0.022	0.041	0.56	24	0.007		-0.022	0.036	0.63	13	-0.058		-0.136	0.019	0.14	7	0.063		0.019	0.107	0.005
Follow up period (month)	26	-0.027		-0.076	0.022	0.28	27	-0.040		-0.071	-0.008	0.014	13	-0.030		-0.154	0.094	0.63	9	-0.037		-0.075	0.001	0.06
Treatment						0.09						0.27						0.96						0.50
Medication or system injection	7		-0.278	-0.662	0.066		10		-0.012	-0.253	0.230		7		-0.478	-1.057	0.102		2		-0.177	-0.813	0.458	
Instillation	8		-0.314	-0.639	0.011		11		-0.282	-0.531	-0.034		3		-0.213	-1.132	0.706		4		-0.628	-1.127	-0.129	
Intravesical injection	8		-0.556	-0.888	-0.224		5		-0.264	-0.650	0.122		1		-0.314	-1.790	1.161		3		-0.305	-0.815	0.205	
Physical therapy	1		-0.660	-1.529	0.208																			
Others	3		0.336	-0.217	0.888		1		0.397	-0.559	1.352		3		-0.523	-1.420	0.373							
Country						0.18						0.14						0.38						0.24
Western	17		-0.38z	-0.612	-0.149		19		-0.079	-0.237	0.080		12		-0.332	-0.713	0.049		4		-0.482	-0.884	-0.079	
Eastern	9		-0.146	-0.471	0.179		7		-0.228	-0.512	0.056		2		-0.767	-1.667	0.134		4		-0.191	-0.534	0.152	
Middle East	1		-1.151	-2.276	-0.025		1		-0.958	-1.882	-0.034								1		-0.933	-1.823	-0.043	
Cystoscopy						0.49						0.92						0.010						
Yes	18		-0.376	-0.614	-0.139		22		-0.146	-0.321	0.028		5		0.065	-0.365	0.495							
No	9		-0.235	-0.562	0.091		5		-0.168	-0.544	0.208		9		-0.685	-1.059	-0.311							
Hunner lesion						0.65						0.68						0.104						0.36
HL+/HL-	6		-0.477	-0.912	-0.041		10		-0.118	-0.393	0.156		3		0.196	-0.441	0.832		3		-0.644	-1.108	-0.179	
HL-	8		-0.371	-0.724	-0.018		5		-0.326	-0.737	0.084		1		-0.314	-1.439	0.810		3		-0.278	-0.705	0.148	
N/A	13		-0.242	-0.520	0.036		12		-0.13	-0.39	0.13		10		-0.62	-1.008	-0.221		3		-0.23	-0.62	0.17	

*k* number of effect sizes, *β* regression coefficient, *SMD* standardized mean difference, *p*-value from meta-regression analysis using the restricted maximum likelihood

### Publication bias

The statistical approaches for the detection of publication bias or a small-study effect are shown in eFigure 7 in the Supplement. Individual symptoms (pain, frequency, urgency and nocturia) showed visual asymmetric graphics in funnel plots, and Egger’s regression also suggested publication bias (all *P* < .05). However, the structured questionnaires (ICPI, ICSI, PUF and FBV) showed symmetry, and Egger’s regression did not support publication bias or a small-study effect (all *P* > 0.05).

### Quality assessment

The seventy studies were evaluated using the five RoB 2.0 domains to determine the risk of bias. In D1, forty-seven studies and twenty-three were classified as “Some concerns” and “Low”. In D2 & D4, one study was classified as “igh”. In D3, nine studies were classified as “Some concerns”. In D5, all studies were classified as “Low”. Based on these evaluations, nineteen studies were classified as “Low”, fifty studies as “Some concerns” and one study (Hsieh et al. 2012) as “High” (eFigure 8 in the Supplement).

## Discussion

Through our research, we have revealed for the first time that it is appropriate to try a combination of multiple treatments clinically rather than just one treatment in treating the difficult-to-overcome disease called interstitial cystitis. Even though the outcomes of the NMA were in keeping with the data published in the Cochrane review [9], the former highlighted an alternative perspective in that in comparison to a placebo, the overall effects of treatment for discomfort, nocturia, ICPI and ICSI were greater. The types of therapy applied included intravesical instillation or injection, and additional strategies, such as diet modification and hyperbaric oxygen. The meta-regression analysis demonstrated that for frequency, the longer the follow-up duration, the more improved the clinical results. Ageing was identified as a negative moderator associated with a poorer end result in nocturia. The NMA showed that in comparison to a placebo, no single therapy offered any benefit to patients with IC. However, where more than one therapeutic approach was applied, such as an adjunctive immune modulator, intravesical injection, intravesical instillation with BTX or HA, or HA and CS, or changes in diet, more positive outcomes could be anticipated which could be demonstrated in the future by RCTs.

The study NMA objective was to evaluate the effectiveness of any drug treatments available for IC. Although the underlying causes of IC remain poorly defined, there are some data to suggest that in this condition, there is an aberrant immune response. Thus, controlling the intravesical immune response could be a potential therapeutic strategy.

Injection into the bladder was also categorized during this work. Out of a range of possible treatments, intravesical instillation or injection was shown to be an efficacious therapy in pairwise meta-analysis. Recommendations published by the American Urological Association (AUA) state that this procedure should be performed following the failure of first-line approaches and alongside oral treatments and physiotherapy [3].

Since IC is a chronic condition, therapy and monitoring should be carried out over the long term [19]. Current therapeutic options may not offer a cure; however, in a number of patients, there may be spontaneous recovery, ultimately leading to complete relief from symptoms [3, 19]. An adverse prognostic factor for IC is age. Additionally, increased frequencies of diabetes mellitus, high blood pressure, kidney pathologies, coronary artery disease and raised serum lipids were identified [20]. Psychological symptoms were also demonstrated to be more common in this cohort, with anxiety and depression recognized in 14–52% and 16–70% of patients with IC, respectively [21]. Frequent monitoring and therapy were observed over the preceding five years in 76.3% of patients studied by Yeh et al. [22]. This group had a higher prevalence of chronic pathologies, which could impact the ongoing degree of IC. The majority of individuals who were under frequent review and who had been offered a range of therapies continued to present with bladder discomfort symptoms and were seeking novel therapeutic options. Those who no longer attended review appointments during the preceding five years had been less symptomatic following their early therapy. The current meta-regression analysis demonstrated that the more prolonged the follow-up period, the more likely frequency was to improve. Advancing years were associated with poorer clinical endpoints, e.g. nocturia; in general, each additional year of age was associated with a 3.4% deterioration in symptom score.

Although a single treatment study has been reported that BTX is effective in pain relief and that PPS helps improve subjective symptoms in patients [23, 24], the strength of the current NMA is that a de novo combination of therapy is proposed. Despite the lack of statistical evidence for the efficacy of each therapy alone in comparison to a placebo, the therapy with the highest rank for its modality was indicated by the SUCRA ranking analysis. Therapeutic combinations which are anticipated include the use of an immune moderator, injections or instillations into the bladder, e.g. with BTX, HA, or HA and CS, and diet modification.

Immune cells liberate tumor necrosis factor-alpha (TNF-α), a proinflammatory cytokine that has been implicated in the inflammatory responses associated with IC. In urothelial cells from the bladder of individuals with ulcerative IC, amplified expression of TNF-α has been observed. Additionally, serum TNF-α titers, as well as levels of additional proinflammatory cytokines, are higher in patients with IC than in control subjects. These data suggest that in addition to the activation of mast cells, these cytokines are key inflammatory mediators in the disease processes underlying IC [25].

In vivo models of autoimmune cystitis have been used to experimentally show that inhibition of TNF-α diminishes inflammation within the bladder by disrupting the triggering of mast cells [26–28]. In this context, vesical inflammation related to IC and the associated symptoms could be relieved by TNF-α inhibition and suppression of mast cell activation using agents that oppose the actions of TNF-α, e.g. adalimumab and certolizumab pegol. Clinical endpoints were enhanced following therapy with adalimumab in individuals with a moderate to severe degree of discomfort from IC [29]. However, as the placebo effect was significant, a positive proof of concept with respect to adalimumab could not be demonstrated. A further study documented that the use of certolizumab pegol in female subjects with moderate to severe unremitting IC offered greater symptom relief than a placebo [30].

In the course of development, nerve growth factor (NGF) is a key requisite for sensory and sympathetic nerve cell survival. It has also been determined that NGF is a peripheral mediator in a number of inflammatory disorders associated with pain [31]. As a humanized monoclonal antibody against NGF, tanezumab has a high affinity and specificity for bonding with NGF, thus blocking the mediator from engaging with nociceptive nerve cell receptors. The latter comprises the high- and low-affinity receptors, tropomyosin-related kinase A and p75 [32]. Following the use of tanezumab, a clinical trial reported an improvement in pain scores as well as enhanced function and overall evaluation outcomes in individuals with chronic painful disorders, not including IC [33–35]. The initial effectiveness of pain relief following pain therapy in IC was documented by Evans et al. [36], but these data were too ambiguous to be incorporated within the current meta-analysis or NMA.

Fulranumab is a human recombinant monoclonal antibody and a powerful human NGF inhibitor. A positive dose effect has been demonstrated in painful disorders, including the neuropathy associated with diabetes. Additionally, mixed data regarding its efficacy in comparison with a placebo have been presented. In patients with osteoarthritic symptoms, fulranumab demonstrated greater efficacy than an opiate, although, in low back pain, no benefit over a placebo was seen [32]. A study by Wang et al. [37] failed to show any positive effects from one dose, but the sample size was restricted and the study had insufficient statistical power. The clinical observations from these studies suggest that it would be valuable to carry out a clinical study in larger or more selected populations to establish whether fulranumab would be effective as a treatment for the refractory pain experienced by patients with IC. Additional possible treatment strategies include options from complementary and alternative medicine. These include behavioural and physical therapies approaches to ameliorate stress and modification of the diet [4, 6]. In patients who were symptomatic with IC, an intense and systematic alteration of the diet was associated with symptom relief within three months; this benefit continued for a minimum of 12 months [38].

There are several limitations associated with this study. Firstly, despite the numerous studies encompassed, there is a surplus of specific therapeutic strategies and the study populations for the individual approaches are small. Secondly, the only first-line therapy evaluated was diet modification. AUA guidelines suggest that patient education, patient self-efficacy, behavioural changes and stress reduction should be first-line therapies in individuals with IC [3]. Finally, the large number of therapeutic possibilities evaluated precluded their concurrent analysis in the NMA.

## Conclusions

In patients with IC, therapy is not straightforward. The current work has led to the recognition of a therapeutic combination for this condition that could be anticipated to have a positive clinical outcome. It was established that ongoing therapy is of value, and that the efficacy of treatments is diminished in individuals of advancing years. Additional RCTs are required to ascertain the efficacy of combined therapeutic approaches, such as immunomodulatory compounds, intravesical injection or instillation with BTX or HA, or HA and CS and diet modification.

### Supplementary Information

**Supplementary Material 1.**

## Data Availability

Data are publicly available. The data supporting this study’s findings are available from the corresponding author upon reasonable request.
