# Supplementary material for: Current updates relating to treatment for interstitial cystitis/bladder pain syndrome: systematic review and network meta-analysis
Source: BMC Urol. 2024 Apr 24;24:95. doi: 10.1186/s12894-024-01485-w (PMC11040764; doi:10.1186/s12894-024-01485-w)
Supplement: Supplementary file 1 — Supplementary Material 1. [file 12894_2024_1485_MOESM1_ESM.docx]

**Supplementary appendix**

Park JJ, Kim KT, Lee EJ, Shim SR, Kim JH. Current updates relating to treatment for interstitial cystitis/bladder pain syndrome: systematic review and network meta-analysis

This supplemental material has been provided by the authors to give readers additional information about their work.

eFigure 1. Network plots, forest plots, and SUCRA values of frequency in network meta-analysis.

eFigure 2. Network plots, forest plots, and SUCRA values of urgency in network meta-analysis.

eFigure 3. Network plots, forest plots, and SUCRA values of nocturia in network meta-analysis.

eFigure 4. Network plots, forest plots, and SUCRA values of ICPI in network meta-analysis. ICPI = interstitial cystitis problem index.

eFigure 5. Network plots, forest plots, and SUCRA values of ICSI in network meta-analysis. ICSI = interstitial cystitis symptom index.

eFigure 6. Network plots, forest plots, and SUCRA values of FBV in network meta-analysis. FBV = functional bladder volume.

eFigure 7. Network funnel plots for publication bias.

eFigure 8. Risk of bias assessment.

eTable 1. Search queries.

eTable 2. Characteristics of studies included in the systematic review.

eTable 3. A reference list of included studies in the systematic review.

eTable 4. Specific treatments and outcomes in the meta-analysis.

eTable 5. Moderator effects of structured questionnaires for interstitial cystitis in pairwise meta-analysis.

eTable 6. Specific meta-analysis methodology.

eTable 7. PRISMA NMA Checklist of Items to Include When Reporting A Systematic Review Involving a Network Meta-analysis.

eFigure 1. Network plots, forest plots, and SUCRA values of frequency in network meta-analysis.


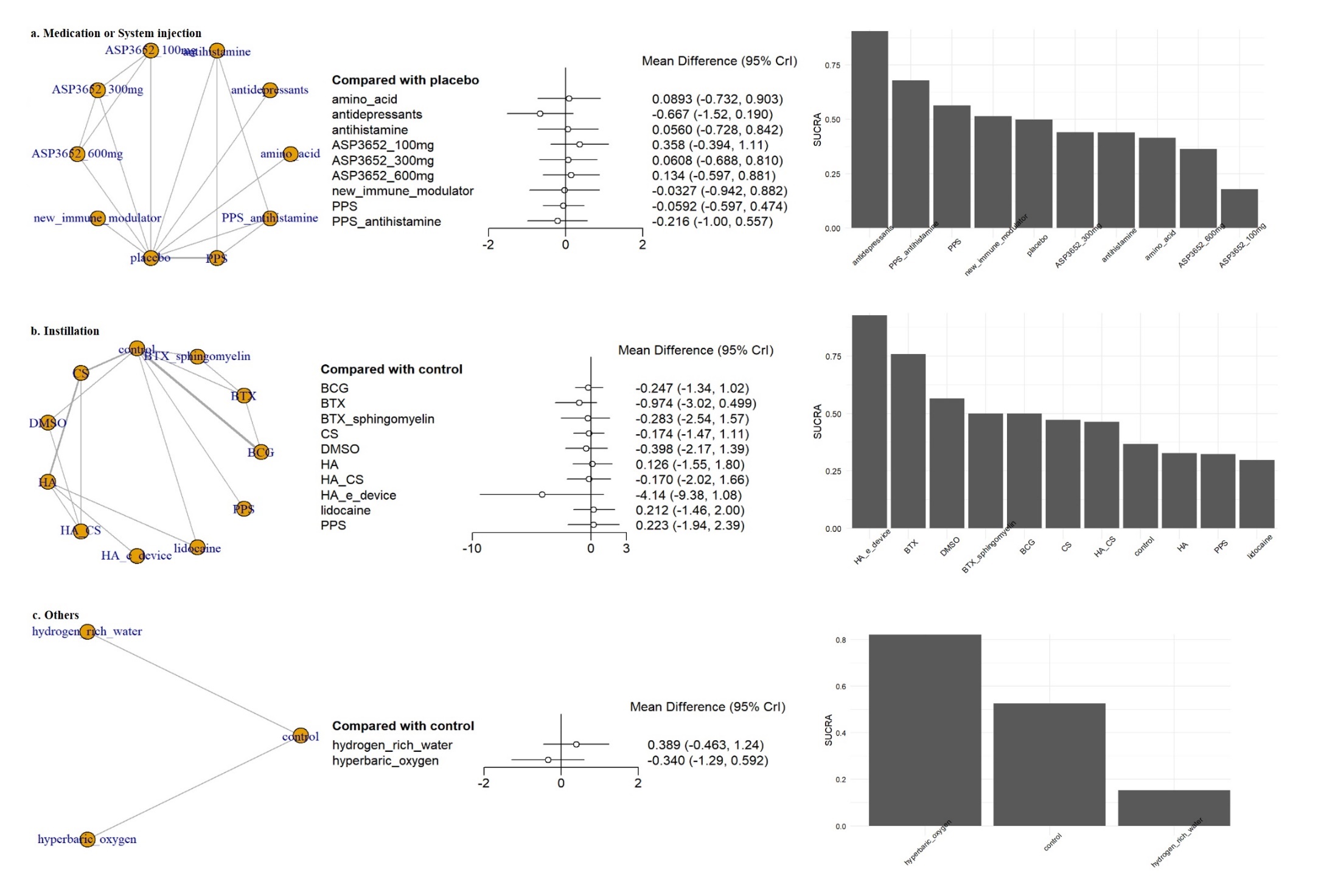


eFigure 2. Network plots, forest plots, and SUCRA values of urgency in network meta-analysis.


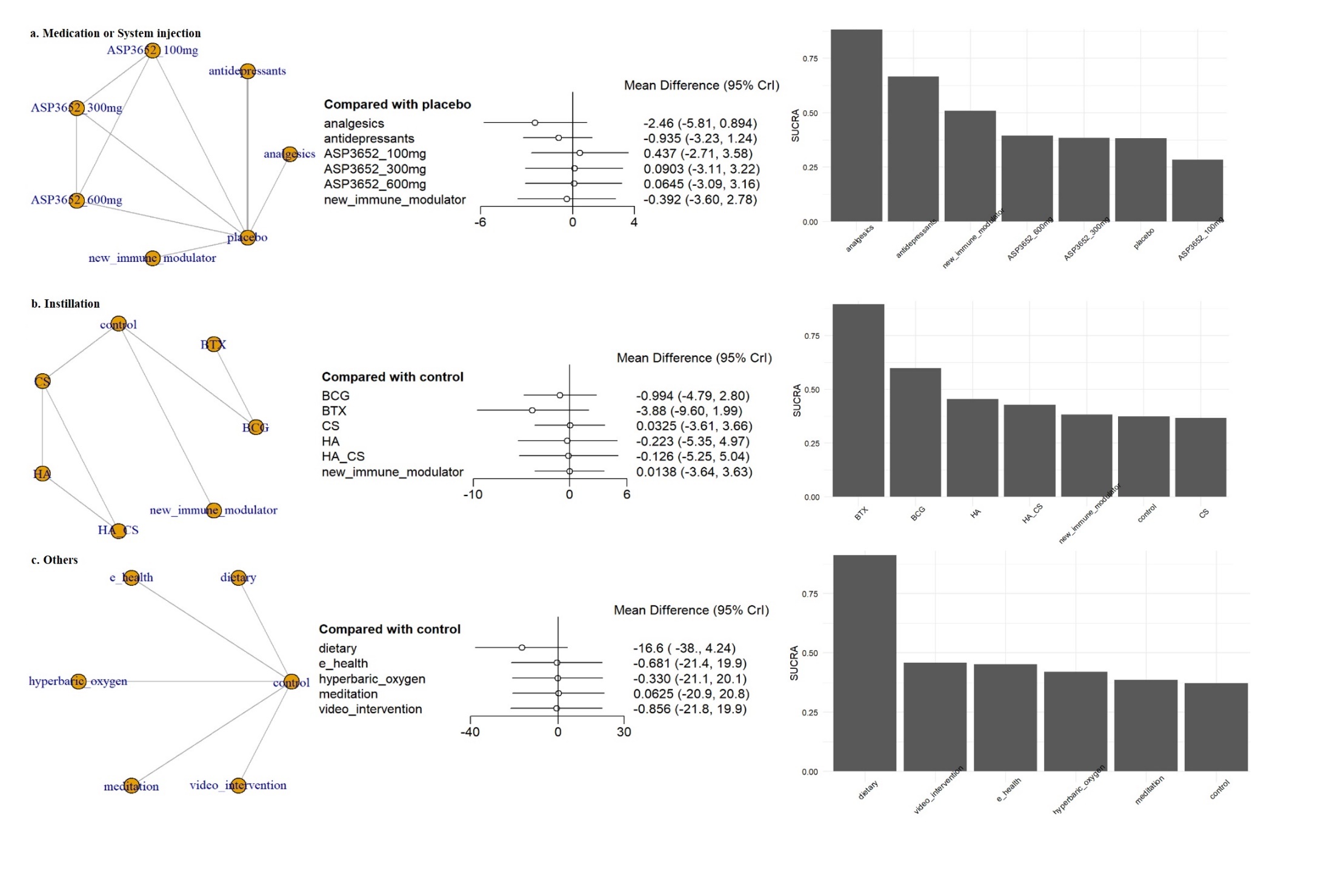


eFigure 3. Network plots, forest plots, and SUCRA values of nocturia in network meta-analysis.


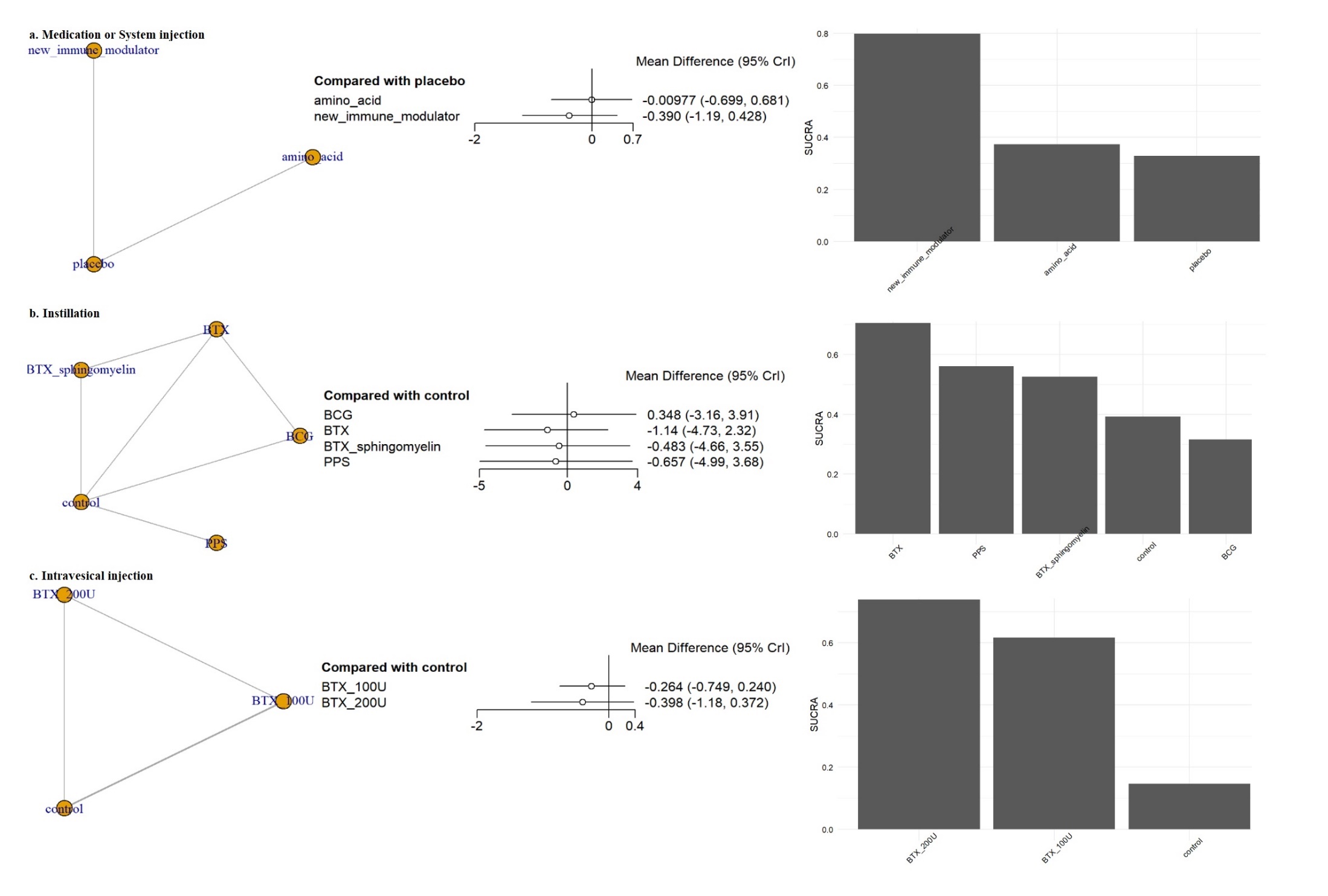


eFigure 4. Network plots, forest plots, and SUCRA values of ICPI in network meta-analysis. ICPI = interstitial cystitis problem index.


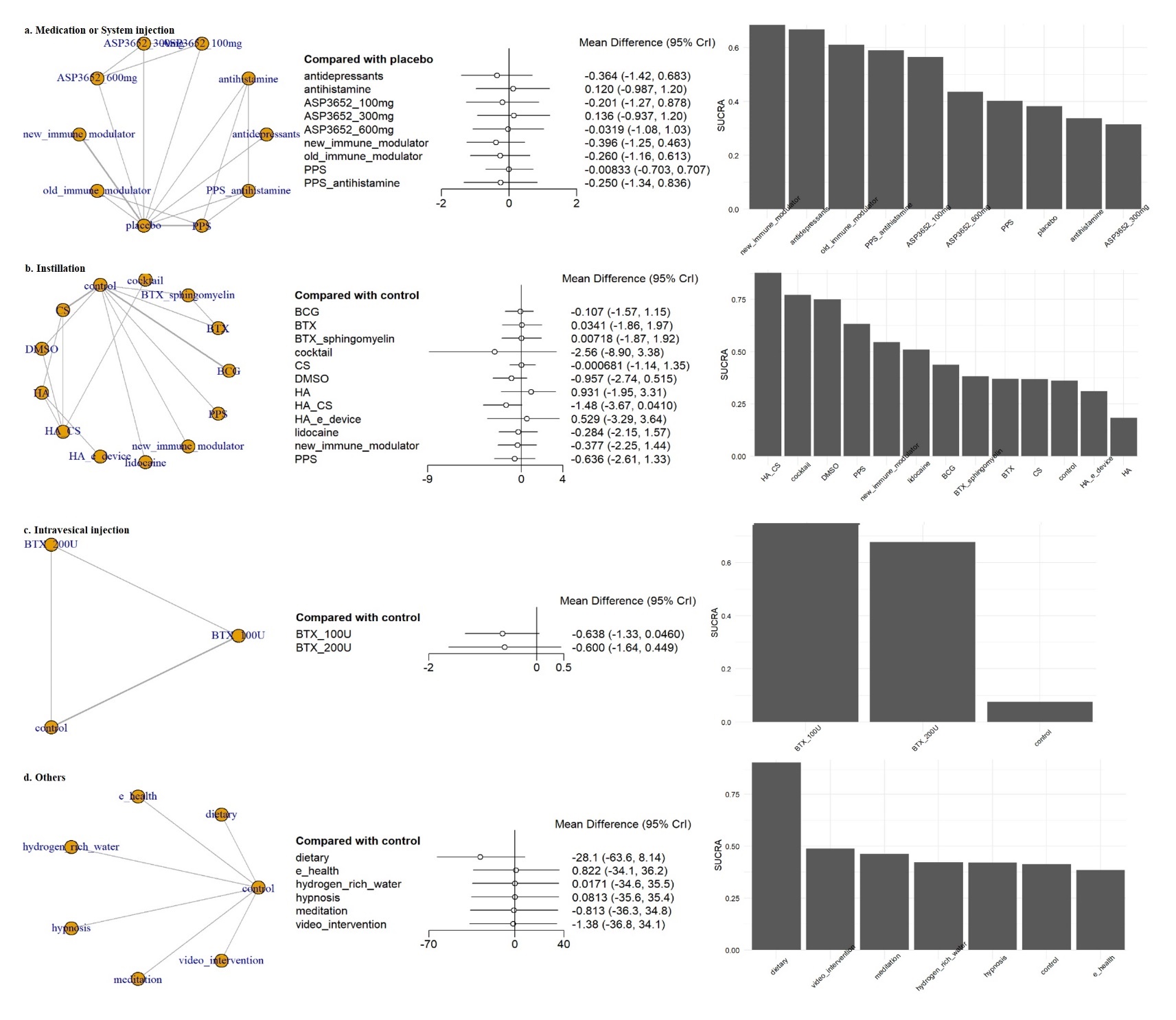


eFigure 5. Network plots, forest plots, and SUCRA values of ICSI in network meta-analysis. ICSI = interstitial cystitis symptom index.


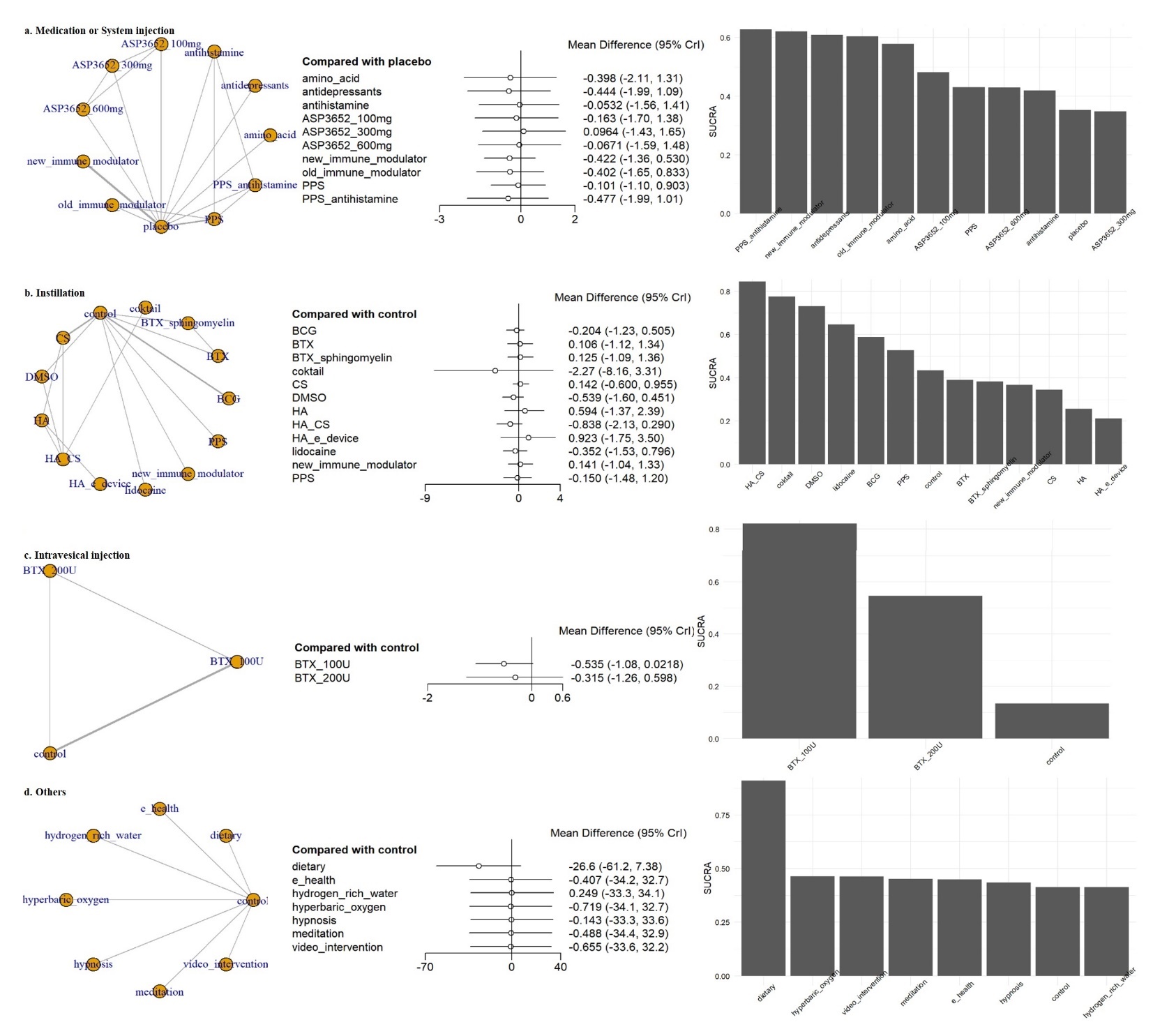


eFigure 6. Network plots, forest plots, and SUCRA values of FBV in network meta-analysis. FBV = functional bladder volume.


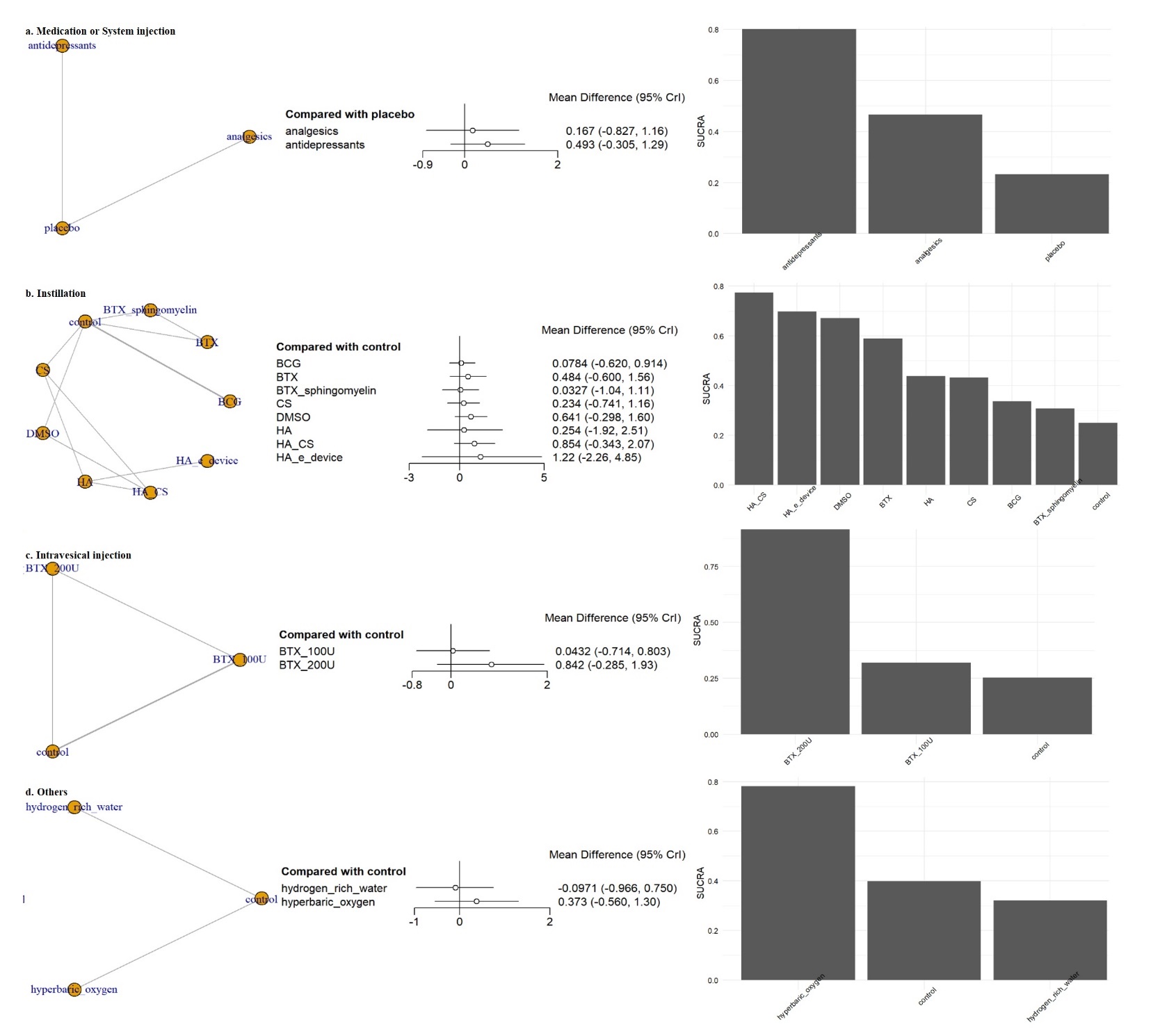


eFigure 7. Network funnel plots for publication bias.


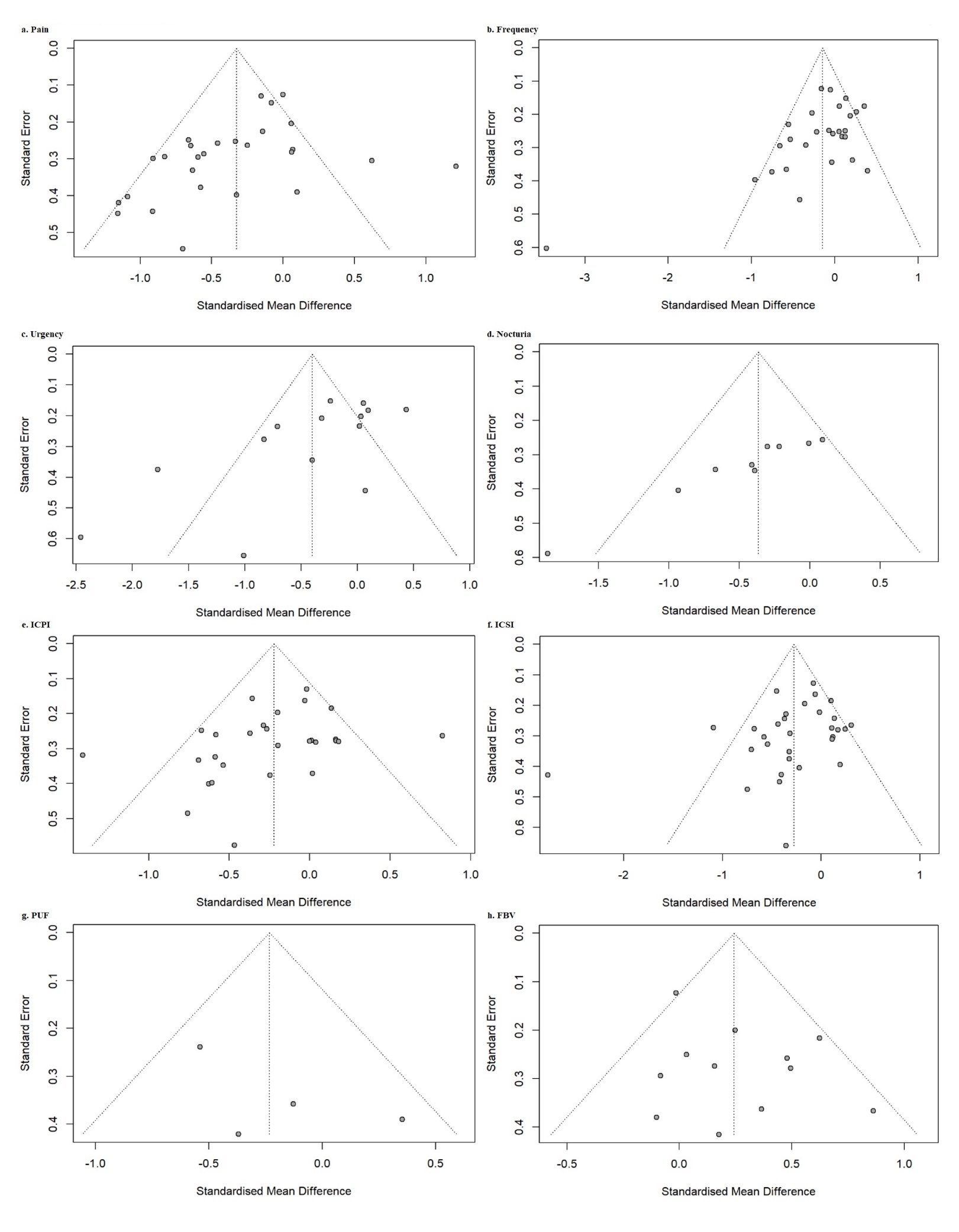


eFigure 8. Risk of bias assessment.


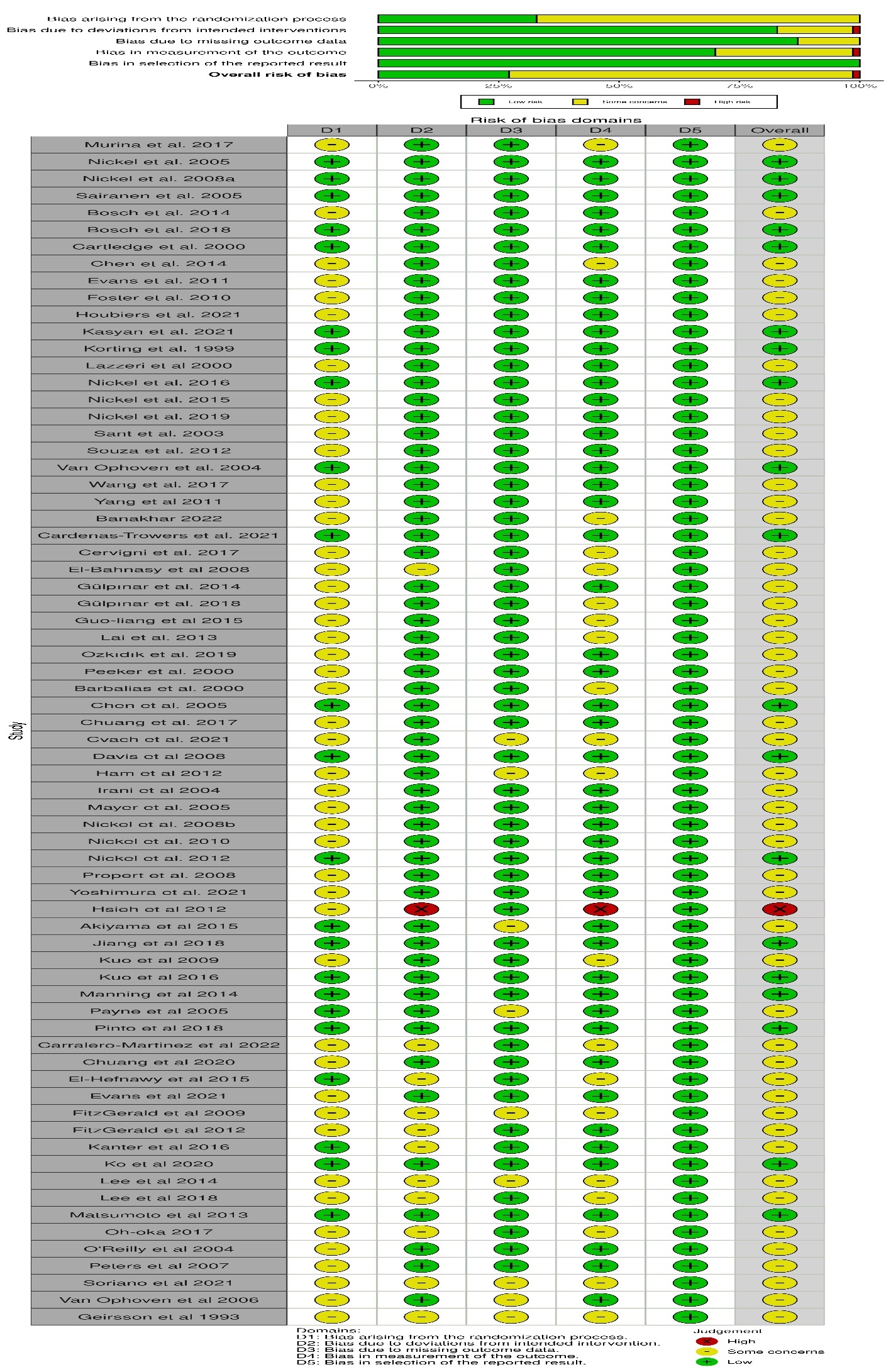


eTable 1. Search queries

| **PubMed and Cochrane Library** | |
| --- | --- |
|  | 1. Disease; colonic polyp  ("cystitis, interstitial"[MeSH Terms] OR "interstitial cystitis"[tiab]) |
|  | 2. Publication type and Species  ("humans"[MeSH Terms] AND "randomized controlled trial"[Publication Type]) |
|  |  |
| **Embase** | |
|  | 1. Disease; colonic polyp  ('interstitial cystitis'/exp OR 'interstitial cystitis':ti,ab) |
|  | 2. Publication type and Species  ('human'/exp AND 'randomized controlled trial'/de) |

eTable 2. Characteristics of studies included in the systematic review

| Study | Region | Study design | Inclusion criteria | Treatment | Number of patients | Mean age (SD) | Follow up period (months) |
| --- | --- | --- | --- | --- | --- | --- | --- |
|  |  |  |  |  |  |  |  |
| Murina et al. 2017 | Western | RCT | Women diagnosed with vestibulodynia/painful bladder syndrome (VBD/PBS) | G1 : amitriptyline,  G2 : amitriptyline plus a commercially available preparation containing ALA 600 mg, DHA 250 mg, and eicosapentaenoic acid 16.67 mg | 84 (G1 : 41, G2 : 43) | G1 : 30.2 (7.3), G2 : 30.0 (8.2) | 2 |
| Nickel et al. 2005 | Western | RCT | Adults with a diagnosis of IC (history of IC symptoms for 6 months or longer, positive cystoscopic examination combined with bladder pain and urgency) | G1 : 100 mg three times daily PPS,  G2 : 200 mg three times daily PPS,  G3 : 300 mg three times daily PPS | 380 (G1 : 128, G2 : 125, G3 : 127) | G1 : 45.0 (19-84), G2 : 43.5 (19-80), G3 : 44.2 (19-81) | 8 |
| Nickel et al. 2008 | Western | RCT | Adults with a diagnosis of IC (history of IC symptoms for 6 months or longer, positive cystoscopic examination combined with bladder pain and urgency | G1 : early treatment (treatment initiation 6 months or less after IC diagnosis),  G2 : late treatment (treatment initiation 24 months or longer after IC diagnosis) | 103 (G1 : 57, G2 : 46) | G1 : 41 (19-77), G2 : 48 (24-84) | 8 |
| Sairanen et al. 2005 | Western | RCT | Patients meeting the NIDDK criteria of interstitial cystitis | G1 : cyclosporine (3 mg/kg divided into 2 daily),  G2 : PPS (100 mg 3 times daily) | 64 (G1 : 32, G2 : 32) | G1 : 56.2 (14.7), G2 : 59.7 (13.0) | 6 |
| Bosch et al. 2014 | Western | RCT | Men and women 18 to 65 years old, previously diagnosed with moderate or severe IC/BPS | G1 : 80 mg loading dose of subcutaneous adalimumab followed by 40 mg every 2 weeks,  G2 : subcutaneous placebo | 43 (G1 : 21, G2 : 22) | G1 : 45.2 (14.0), G2 : 46.5 (13.4) | 1,2,3 |
| Bosch et al. 2018 | Western | RCT | Women aged 18–65 yr with moderate to severe IC/BPS as defined with scores of 7 on the O’Leary-Sant Interstitial Cystitis Symptom Index (ICSI) | G1 : subcutaneous certolizumab pegol 400 mg at 0, 2, 4, 8 weeks,  G2 : placebo (sterile saline) at 0, 2, 4, 8 weeks | 42 (G1 : 28, G2 : 14) | Median (IQR) G1 : 50 (37-56), G2 : 54 (38-60) |  |
| Cartledge et al. 2000 | Western | RCT | Any patient ful®lling the diagnostic criteria for IC established by the NIADDK (Glomerulations (petechial haemorrhage) or Hunner's ulcer on endoscopy and pain associated with the bladder or urinary urgency) | G1 : L-arginine (2.4 g/day) for one month,  G2 : placebo for one month | 16 | mean (range) 51 (26-76) | 1 |
| Chen et al. 2014 | Eastern | RCT | Women with 12-48 months of characteristic symptoms of IC/PBS according to the National Institute of Diabetes and Digestive and Kidney Diseases criterion | G1 : daily low-dose sildenafil (25 mg; Pfizer),  G2 : placebo (starch) | 48 (G1 : 24, G2 : 24) | G1 : 38.3 (5.4), G2 : 37.8 (4.4) | 3 |
| Evans et al. 2011 | Western | RCT | Patients with moderate to severe IC defined as scores of 13 or greater on the Pelvic Pain and Urgency/Frequency symptom questionnaire and 7 or more on the O’Leary-Sant ICSI | G1 : single IV dose of 200 µg/kg tanezumab,  G2 : placebo | 52 (G1 : 29, G2 : 23) |  | 4 |
| Foster et al. 2010 | Western | RCT | Patients at least 18 years of age, who reported both bladder pain/discomfort and urinary frequency of 3 or greater on separate 0–10 Likert scales over the previous 4 weeks | G1 : Amitriptyline (dose was increased on a weekly basis from 10mg to 75mg, then maintained at the highest tolerable dose until 12 weeks) with standardized education and behavioral modification program,  G2 : placebo with standardized education and behavioral modification program | 271 (G1 : 135, G2 : 136) | G1 : 38.0 (13.8), G2 : 39.9 (14.0) | 3 |
| Houbiers et al. 2021 | Western | RCT | Female patients with IC/BPS with moderate to severe pain | Comparison of effects according to dose of ASP3652 (fatty acid amide hydrolase (FAAH) inhibitor) G1 : ASP3652 50mg bid, G2 : ASP3652 150mg bid, G3 : ASP3652 300mg bid, G4 : placebo | 286 (G1 : 53, G2 : 55, G3 : 96, G4 : 82) | G1 : 49.6 (14.8), G2 : 53.5 (16.6), G3 : 50.3 (16.8), G4 : 51.4 (15.8) | 3 |
| Kasyan et al. 2021 | Western | RCT | Patients with diagnosis of bladder pain syndrome/interstitial cystitis, aged 18–55 years, had a ICSI score at lease 8, bladder pain must have presented for longer than 6 months, Endoscopic criteria : petechial hemorrhages or Hunner's lesions of the bladder | G1 : Pentosane polysulfate 100 mg tid,  G2 : placebo 100 mg tid | 89 (G1 : 45, G2 : 44) | G1 : 36.93 (10.60), G2 : 35.43 (10.15) | at the end of treatment |
| Korting et al. 1999 | Western | RCT | Patients with interstitial cystitis who consented to participate in this study | G1 : L-arginine 500 mg tid with usual care for 3 months,  G2 : placebo tid with usual care for 3 months | 53 (G1 : 27, G2 : 26) | G1 : 46.6 (14.3), G2 : 52.7 (13.6) | 3 |
| Lazzeri et al 2000 | Western | RCT | men and women with hypersensitive disorder and severe bladder pain. Frequency greater than 8 voids, nocturia greater than 2 voids, daily urgency and bladder pain for at least 6 months. Furthermore, we considered absent urinary tract infection, including tuberculosis, within the last 3 months; absent functional disorders of the lower urinary tract as detrusor overactivity; and no calculi or proliferative vesical pathology | G1 : resiniferatoxin (RTX) 10 im in 0.1% ethanol G2 : NaCl | 18 (G1 : 9, G2 : 9) | 43.4 | 1, 3 |
| Nickel et al. 2016 | Western | RCT | Women aged 18 to 75 years old with a diagnosis of IC/BPS for greater than 6 months but 15 years or less and bladder pain for 12 months or more | Comparison of effects according to dose of AQX-1125 (SH2-containing inositol-50-phosphatase 1 activator)  G1 : AQX-1125 200mg daily,  G2 : placebo | 69 (G1 : 37, G2 : 32) | G1 : 52.1 (14.9), G2 : 53.1 (12.9) | 6 wks |
| Nickel et al. 2015 | Western | RCT | Women aged 18 years old or older with IC/BPS based on a total score of 8 or greater on ICSI, and with an average of at least 10 voids per day | G1 : PPS 100 mg QD,  G2 : PPS 100 mg TID (the FDA approved dose),  G3 : placebo | 368 (G1 : 128, G2 : 122, G3 : 118) | G1 : 45.6 (15.73), G2 : 42.7 (15.71), G3 : 44.6 (14.58) | 6 |
| Nickel et al. 2019 | Western | RCT | 18 to 80 years old patient with a diagnosis of IC/BPS for more than 6 months | Comparison of effects according to dose of SH2-containing inositol-5'-phosphatase 1 (SHIP1) activator  G1 : SHIP1 activator 100mg daily for 12 weeks,  G2 : SHIP1 activator 200mg daily for 12 weeks,  G3 : placebo | 341 (G1 : 114, G2 : 113, G3 : 114) | G1 : 50.1 (15.17), G2 : 49.7 (14.84), G3 : 4737 (15.10) | 3 |
| Sant et al. 2003 | Western | RCT | Age ≥ 18,  diagnosis of IC confirmed by cystoscopy and hydrodistention,  moderate symptoms of urinary frequency (at least 11 times daily), pain/discomfort (at least 4 on a 0 to 9 Likert scale) for at least 24 weeks | G1 : PPS (100 mg tid),  G2 : Hydroxyzine (10-50mg daily),  G3 : combination therapy (PPS (100 mg tid) + Hydroxyzine (10-50mg daily)),  G4 : Placebo | 121 (G1 : 31, G2 : 29, G3 : 30, G4 : 31) | G1 : 47.8 (13.9), G2 : 48.7 (15.1), G3 : 43.7 (15.1), G4 : 41.0 (15.5) | 6 |
| Souza et al. 2012 | Western | RCT | Patients with IC/PBS diagnosed on the basis of pelvic pain, pressure, or discomfort perceived to be related to the urinary bladder accompanied by at least one urinary symptom, such as urgency or frequency | G1 : Cystex® capsule (Acriflavin hydrochloride 15.00 mg; Methenamine 250.00 mg; Methylene blue 20.00 mg; Beladona extract 15.00 mg) for 21 days,  G2 : placebo for 21 days | 22 (G1 : 11, G2 : 11) | G1 : 54.9 (5.1), G2 : 55.3 (5.8) | 1 (3wks) |
| Van Ophoven et al. 2004 | Western | RCT | Patients who all met the symptom criteria of the National Institute of Diabetes, Digestive and Kidney Diseases for IC | G1 : amitriptyline 25-100mg at bedtime (maximum allowed dosage),  G2 : placebo | 50 (G1 : 25, G2 : 25) | G1 : 50.5 (14.4), G2 : 60.2 (17.5) | 4 |
| Wang et al. 2017 | Western | RCT | Patients aged 18 to 80 years with IC/BPS based on the validated O’Leary-Sant interstitial cystitis symptom index (ICSI) and chronic bladder pain for at least 6 months, accompanied by urinary urgency, urinary frequency (≥8 voids daily), and/or nocturia. | G1 : fulranumab 9 mg injected subcutaneously (SC) into the thigh once every 4 weeks,  G2 : placebo injected subcutaneously (SC) into the thigh once every 4 weeks | 31 (G1 : 14, G2 : 17) | G1 : 50.6 (10.68), G2 : 46.2 (13.56) | 3, 6 (26 wks) |
| Yang et al 2011 | Western | RCT | men and women older than 18 years. Eligibility required fulfilment of all of certain criteria, including (1) persistent symptoms of urinary frequency and pain rated at least 4 on a scale of 0 to 10, (2) failure of at least 24 weeks of active treatment with a minimum of 3 standard forms of therapy or combination of therapies for IC/PBS, (3) cystoscopic diagnosis of IC/PBS in the past with findings of glomerulations and/or ulcerations and (4) screening cystoscopy within the 24 weeks before study entry to evaluate for an unevaluated pathological condition | G1 : immunosuppressant 1 gram daily, mycophenolate mofetil (MMF) for 14 days, then 1 gram BID for 10 weeks G2 : placebo | 58 (G1 : 39, G2 : 19) | G1 : 51.3 (10.3), G2 : 51.8 (11.6) | 4 |
| Banakhar 2022 | middle eastern | RCT | Patients who were diagnosed as IC/BPS. Diagnosis of IC/BPS was made based on the clinical symptoms history of bladder pain and discomfort related to bladder filling accompanied by frequency in the absence of infection; Patients meeting the NIDDK (National Institute of Diabetes, Digestive and Kidney Diseases) criteria for IC; both ulcerative and non-ulcerative types | All study group patients underwent bladder hydrodisten- tion, additional ulcer fulgurations for ulcerative IC. On the second day after hydrodistention, both group received intravesical therapy;  G1 : cocktail intravesical therapy (NaOH + heparin + lidocaine),  G2 : Ialuril (HA and CS) | 24 (G1 : 18, G2 : 6) | G1 : 41.72 (15.578), G2 : 38.50 (12.566) | 3 (14 wks) |
| Cardenas-Trowers et al. 2021 | Western | RCT | Women aged 18 years and older with a clinical diagnosis of interstitial cystitis–bladder pain syndrome; 6 points or higher on either the problem or symptom index of the O’Leary- Sant Questionnaire | 6 bladder instillations (heparin, 2% viscous lidocaine, 8.4% sodium bicarbonate, and 0.5% bupivacaine);  G1 : with triamcinolone acetonide (triamcinolone acetonide; 1 vial, 40 mg/mL), G2 : without triamcinolone acetonide | 90 (G1 : 45, G2 : 45) | G1 : 46.6 (14.0), G2 : 37.3 (13.3) | 1 (3 wks), 2 (6 wks) |
| Cervigni et al. 2017 | Western | RCT | Female patients aged 18 years or more with a diagnosis of BPS/IC, according to the European Society for the Study of IC/PBS (ESSIC) Criteria; presence of pain with at least one other urinary symptom for at least 6 months | G1 : 13 weekly instillations of HA (1.6%) and CS (2.0%) (Ialuril®; IBSA),  G2 : 13 weekly instillations of 50% DMSO solution (RIMSO®; Bioniche) | 100 (G1 : 74, G2 : 36) | G1 : 50.95 (14.97), G2 : 48.78 (17.70) | 3, 6 |
| El-Bahnasy et al 2008 | Western | RCT | female patients who met the NIDDK criteria for IC/PBS and reported at least moderate pain and frequency for minimum of 6 months | G1 : intravesical BCG administration in the form of weekly instillations G2 : intravesical injections of BTX-A under local anesthesia in the form of intraurethral 2% lidocaine jell and intravesical 2% lidocaine on 100 ml saline for 10 minutes | 32 (G1 : 16, G2 : 16) | - | 6 |
| Gülpınar et al. 2014 | middle eastern (Turkey) | RCT | Patients with diagnosis of BPS/IC; fulfilled the National Institute of Diabetes and Digestive and Kidney Diseases criteria for BPS/IC | G1 : 40mg hyaluronic acid intravesical instillation and retaining for at least 60 mins,  G2 : 40mg hyaluronic acid and electromotive drug administration (EMDA) intravesical instillation and retaining for at least 60 mins. (using the generator) | 31 (G1 : 15, G2 : 16) | G1 : 43.5 (4.2), G2 : 42.8 (4.0) | 1, 6, 12, 24 |
| Gülpınar et al. 2018 | middle eastern (Turkey) | RCT | Women with diagnosis of BPS/IC; complaints of chronic pelvic pain; voiding frequency ≥ 8 times/24 h, nocturia ≥2 times per night or persistant urge for at least 24 weeks; average pain score of ≥4 (VAS; 0 no pain, 10 unbearable pain) | G1 : intravesical therapy with 50 mL/120 mg sterile sodium hyaluronic acid (HA),  G2 : intravesical therapy with 40 mL/80 mg sodium chondroitin suphate (CS) | 42 (G1 : 21, G2 : 21) | G1 : 48.9 (17.18), G2 : 47.1 (10.7) | 6 |
| Guo-liang et al 2015 | Eastern | RCT | patients with interstitial cystitis | G1 : intravesical hyaluronic acid (HA) after bladder hydrodistention G2 : intravesical heparin sodium-lidocaine (HL) after bladder hydrodistention | 24 (G1 : 13, G2 : 11) | G1 : 45.0 (8.8), G2 : 44.1 (6.7) | 6, 9, 12 |
| Lai et al. 2013 | eastern | RCT | Patients with diagnosis of IC/PBS (followed the East Asian guideline, characteristic symptoms of suprapubic pain, accompanied by urinary frequency, nocturia and cystoscopic findings of glomerulations) | G1 : four weekly intravesical instillations of 40 mg of hyaluronic acid (HA) followed by 5-monthly HA instillations,  G2 : 12 intravesical instillations of 40 mg of HA every 2 weeks for 6 months | 60 (G1 : 30, G2 : 30) | 47.9 (12.9) | 1, 3, 6 |
| Ozkıdık et al. 2019 | middle eastern (Turkey) | RCT | Patients with definitive diagnosis of BPS | G1 : intravesical instillation of hyaluronic acid (HA),  G2 : intravesical instillation of chondroitin sulphate (CS) instillation,  G3 : intravesical instillation of combination of HA and CS instillation | 72 (G1 : 24, G2 : 24, G3 : 24) | G1 : 37.1, G2 : 37.4, G3 : 37.2 | 24 |
| Peeker et al. 2000 | Western | RCT | Patients were diagnosed according to the National Institutes of Health National Institute for Diabetes and Digestive and Kidney Diseases criteria and classified into 2 different groups of interstitial cystitis based on clinical, endoscopic and histopathological criteria | G1 : intravesical instillation of BCG (in 6 weekly instillations of 5 3 108 colony forming units),  G2 : intravesical instillation of dimethyl sulfoxid (in 6 weekly instillations of 50 ml dimethyl sulfoxide solution, 500 mg per ml) | 15 (G1 : 15, G2 : 15),  6 drop out in washiout period | - | 3 |
| Barbalias et al. 2000 | Western | RCT | Women with a mean age of 45 years (range 26 to 69) with a diagnosis of interstitial cystitis according to the inclusion or exclusion criteria of the National Institute of Arthritis, Diabetes, Digestive, and Kidney Diseases | G1 : gradual intravesical instillation of saline oxybutynin solution,  G2 : gradual intravesical instillation of simple saline | 36 (G1 : 24, G2 : 12) | 45 (26-69) | 1, 3, 6 |
| Chen et al. 2005 | Western | RCT | Patients diagnosed with IC for a minimum of 9 months, based on NIH-NIADDK criteria | G1 : intravesical instillation of 10% ethanol in saline and 0.05 lM resiniferatoxin (RTX),  G2 : intravesical instillation of 10% ethanol in saline and 0.10 lM RTX,  G3 : intravesical instillation of 10% ethanol in saline and placebo | 22 (G1 : 10, G2 : 8, G3 : 4) | G1 +G2 : 43.7, G3 : 56.6 | 1, 3 |
| Chuang et al. 2017 | Eastern | RCT | Patients 20 years old or older with IC/BPS in whom at least 6 months of conventional treatments had failed (The diagnosis of IC/BPS was established based on characteristic symptoms and cystoscopic findings of glomerulations, petechiae or mucosal fissures upon hydrodistention in the last year) | G1 : intravesical instillation of lipotoxin (onabotulinumtoxinA 200 U with 80 mg sphingomyelin),  G2 : intravesical instillation of onabotulinumtoxinA 200 U in normal saline,  G3 : intravesical instillation of normal saline alone | 90 (G1 : 31, G2 : 28, G3 : 31) | G1 : 53.9 (12.9), G2 : 47.8 (9.9, G3 : 55.9 (8.6) | 1 |
| Cvach et al. 2021 | Western | RCT | Women aged 18–80 years old with a diagnosis of BPS/IC in whom oral medical management had failed to control symptoms (diagnosis of BPS/IC based on clinical criteria as outlined in the American Urological Association guidelines) | G1 : cystoscopy and intravesical instillation Clorpactin (0.4% solution, 2 g in 500 ml of normal saline),  G2 : cystoscopy and hydrodistension (≤ 500 ml of normal saline alone under 80 cmH2O pressure for 4 min) | 50 (G1 : 25, G2 : 25) | G1 : 52 (41-67), G2 : 61 (32-70) | 1, 3 |
| Davis et al 2008 | Western | RCT | Females who were older than 18 years, diagnosed with IC within 1 year of the beginning of the study, and previously untreated with either intravesical or oral PPS | G1 : Intravesical PPS (twice a week) during the first six weeks + oral PPS (200mg/twice a day) during the entire period of the study G2 : Intravesical placebo during the first six weeks + oral PPS (200mg / twice a day) during the entire period of the study | 41 (G1 : 21, G2 : 20) | median G1 : 36.9 (31.9, 45.1), G2 : 38.7 (26, 42.7) | 1 (6 wks), 3 (12 wks), 5 (18 wks) |
| Ham et al 2012 | Eastern | RCT | women with IC refractory to classic medical treatment | G1 : 50 mL resiniferatoxin (RTX) in 10% ethanol + hydrodistension G2 : hydrodistension | 18 (G1 : 8, G2 : 10) | G1 : 54.5 (9.1), G2 : 56.9 (7.8) | 3 |
| Irani et al 2004 | Mideastern | RCT | patients who met NIDDK diagnostic criteria were selected from among 150 females with irritative symptoms and bladder pain, after performing cystoscopy under anesthesia, bladder hydrodistention, and other preliminary evaluations | G1 : intravesical installation of 120 mg of BCG vaccine through foley catheter for six weeks G2 : intravesical installation of 50 cc of normal saline (as placebo) through foley catheter for six weeks | 30 (G1 : 15, G2 : 15) | G1 : 40.8 (13.96), G2 : 36 (12.84) | 24 |
| Mayer et al. 2005 | Western | RCT | Patient at least 18 years old and received a diagnosis of IC, confirmed by cystoscopy and hydrodistention, following the National Institutes of Health-NIDDK criteria (urinary frequency and pain/discomfort for at least 24 weeks before study entry) | G1 : intravesical instillation of BCG up to 6 times within 6 to 10 weeks,  G2 : intravesical instillation of placebo up to 6 times within 6 to 10 weeks | 265 (G1 : 131, G2 : 134) | G1 : 48.1 (13.9), G2 : 47.3 (13.2) | 8 |
| Nickel et al. 2008 | Western | RCT | Patients aged 18–75 years with a history of symptoms of bladder pain/discomfort of ≥ 4 on a 10-point Likert scale, suprapubic pain related to bladder filling, and frequency | G1 : intravesical instillation of PSD597 (a patented combination of 200 mg lidocaine, alkalinized with a sequential instillation of 8.4% sodium bicarbonate solution, to a final volume of 10 mL) once a day for 5 consecutive days,  G2 : intravesical instillation of placebo (10 mL of ordinary saline) once a day for 5 consecutive days | 102 (G1 : 50, G2 : 52) | G1 : 44.5 (13.8), G2 : 49.0 (14.5) | 1 (day 8) |
| Nickel et al. 2010 | Western | RCT | Patients at least 18 years of age with the clinical diagnosis of IC/PBS and no medical condition or therapy that would exclude safe concomitant use of chondroitin sulfate (IC/PBS was diagnosed on the basis of pelvic pain, pressure, or discomfort perceived to be related to the urinary bladder accompanied by at least one other urinary symptom, such as urgency or frequency.) | G1 : intravesical instillation of 20 mL of a sterile 2.0% solution of sodium chondroitin sulfate (Uracyst, Watson Pharmaceuticals, Corona, CA) weekly for 6 weeks,  G2 : intravesical instillation of 20 mL sterile vehicle control solution (the identical phosphate-buffered saline vehicle used for Uracyst) weekly for 6 weeks | 65 (G1 : 33, G2 : 32) | G1 : 45.5 (16.07), G2 : 44.4 (14.87) | 7wk, 12wk |
| Nickel et al. 2012 | Western | RCT | Women diagnosed or rediagnosed with IC/BPS within the previous 2 years (subject-reported average urinary frequency of 8 times/24 hours by a 3-day diary; pain/pressure/discomfort score of 40-80 mm on a pain visual analog scale (VAS); inadequate clinical response after 6 months of conservative treatment) | G1 : intravesical instillation of 20 mL of 2% chondroitin sulfate once weekly for 7 weeks (8 treatments in total),  G2 : intravesical instillation of 20 mL of inactive control (the identical phosphate-buffered saline vehicle used for Uracyst) once weekly for 7 weeks | 98 (G1 : 49, G2 : 49) | G1 : 44.4 (14.59), G2 : 46.8 (14.06) | 3 |
| Propert et al. 2008 | Western | RCT | women and men who had at least moderate symptoms of IC | G1 : up to six intravesical instillations of BCG treatment, over a six to ten week period,  G2 : up to six intravesical instillations of saline (placebo) treatment, over a six to ten week period | 38 (G1 : 22, G2 : 16) | - | 8, 17 |
| Yoshimura et al. 2021 | Eastern | RCT | Japanese IC/BPS patients aged ≥20 years with an ICSI score of ≥9, who exhibited the bladder-centric phenotype of IC/BPS | G1 : intravesical instillation of KRP-116D (50% DMSO solution) every 2 weeks for 12 weeks,  G2 : intravesical instillation of placebo every 2 weeks for 12 weeks | 96 (G1 : 49, G2 : 47) | G1 : 63.6 (14.2), G2 : 64.5 (13.5) | 3 |
| Hsieh et al 2012 | Eastern | RCT | patients with IC according to the criteria of the National Institute of Diabetes, Digestive and Kidney Disease were prospectively included | G1 : hydrodistension plus bladder training (talk with physician, attempt to increase intervoid interval) G2 : hydrodistension (bladder filled with normal saline to maximal capacity at pressure of 80 cmhU) | 70 (G1 : 35, G2 : 35) | G1 : 45.9 (7.8), G2 : 46.1 (7.6) | 6 |
| Akiyama et al 2015 | Eastern | RCT | IC patients who had received HD at least once and medical treatment of one or more oral drugs or intravesical agents, yet remained to be symptomatic | G1 : Immediate injection of BoNT-A 100 U G2 : 1-month delayed injection of BoNT-A 100 U after maintaining the present therapies | 34 (G1 : 18, G2 : 16) | G1 : 64.3 (13.2), G2 : 65.6 (14.6) | 1 |
| Jiang et al 2018 | Eastern | RCT | patients with proven IC/PBS who had failed previous treatments for at least 6 months (A diagnosis of IC/PBS : characteristic symptoms and cystoscopic findings of glomerulations, petechia, or mucosal fissures after hydrodistention) | G1 : intravesical injection of 100 U of BoNT-A (BOTOX, Allergan, Irvine, CA) into the trigone  G2 : intravesical injection of 100 U of BoNT-A (BOTOX, Allergan, Irvine, CA) into the bladder body in the suburothelium | 39 (G1 : 19, G2 : 20) | G1 : 55.1 (9.88), G2 : 53.9 (11.4) | 1, 2 |
| Kuo et al 2009 | Eastern | RCT | Patients with IC/PBS who had failed conventional treatments (diagnosis of IC/PBS had been established based on characteristic symptoms of suprapubic pain related to bladder filling, accompanied by daytime and night-time frequency and cystoscopic findings of glomerulation, petechia, or mucosal ulceration) | G1 : intravesical injection of 200 U of BoNT-A (BOTOX, Allergan, Irvine, CA, USA) G2 : intravesical injection of 100 U of BoNT-A G3 : cystoscopic HD alone | 67 (G1 : 15, G2 : 29, G3 : 23) | - | 3 |
| Kuo et al 2016 | Eastern | RCT | IC/BPS patients who had failed at least 6 months of conventional treatments. Patients who had been treated with at least 2 types of treatment modalities | G1 : intravesical injection of 100U of BoNT-A (BOTOX, Allergan, Irvine,CA) immediately followed by cystoscopic hydrodistention G2 : intravesical injection with normal saline immediately followed by cystoscopic hydrodistention | 60 (G1 : 40, G2 : 20) | G1 : 52.9 (14.3), G2 : 50.2 (13.2) | 2 |
| Manning et al 2014 | Western | RCT | female patients with longstanding refractory IC/PBS (They were followed up for at least 2 years. Study participants all met the National Institute of Diabetes and Digestive and Kidney Diseases (NIDDK) criteria) | G1 : 4-min hydrodistension with injection of 500 U AboBTXA (Dysport®) diluted in 30 ml normal saline G2 : 4-min hydrodistension with injection of 30 ml normal saline into the bladder wall | 53 (G1 : 26, G2 : 27) | G1 : 54, G2 : 53 | 3 |
| Payne et al 2005 | Western | RCT | patients at least 18 years of age with a diagnosis of IC confirmed by visualisation of glomerulations at cystoscopy/hydrodistension, symptoms of bladder pain and urinary urgency for at least 6 months | G1 : single intravesical dose of RTX 0.01 μM G2 : single intravesical dose of RTX 0.05 μM G3 : single intravesical dose of RTX 0.1 μM G4 : single intravesical dose of placebo | 163 (G1 : 43, G2 : 41, G3 : 35, G4 : 44) | G1 : 47.7 (13.1), G2 : 49.7 (13.2), G3 : 47.8 (13.6), G4 : 44.5 (11.9) | 1 |
| Pinto et al 2018 | Western | RCT | women 18 to 60 years old with a diagnosis of BPS/IC for more than 6 months in duration according to ESSIC (European Society for the Study of BPS) criteria | G1 : 10 injections of 1 ml 0.9% sodium chloride containing 10 U OnaBotA (total 10 ml and 100 U, respectively) G2 : 10 injections of 1 ml 0.9% sodium chloride | 19 (G1 : 10, G2 : 9) | G1 : 44.3 (9.9), G2 : 47.4 (11.1) | 1, 2, 3 |
| Carralero-Martinez et al 2022 | Western | RCT | adult patients with a Chronic pelvic pain syndrome (CPPS) diagnosis | G1 : Treatment consisted of 10 capacitive resistive monopolar radiofrequency (CRMRF) sessions G2 : Treatment consisted of 10 unactivated CRMRF sessions | 81 (G1 : 41, G2 : 40) | G1 : 43.8 (14.3), G2 : 43.3 (11.5) | 2 |
| Chuang et al 2020 | Eastern | RCT | Patients with IC/BPS, who aged 20 years or above and had failed at least 6 months of conventional treatments | G1 : extracorporeal shock wave therapy (ESWT) G2 : placebo | 42 (G1 : 21, G2 : 21) | G1 : 55.7 (50.7, 60.7), G2 : 55.6 (49.5, 61.8) | 1, 3 |
| El-Hefnawy et al 2015 | Western | RCT | Patients with complaint suggestive of IC/BPS. Patients who were not improved on pain killers and analgesics after treatment for at least 3 months and were willing to participate in the study. At least 4 weeks elapsed before receiving either line of treatment | G1 : superior hypogastric plexus neurolysis (SHN) block G2 : hydrodistension | 26 (G1 : 14, G2 : 12) | G1 : 32 (6), G2 : 33 (7) | 1 |
| Evans et al 2021 | Western | RCT | women aged ≥18 years with IC/BPS | G1 : a single treatment of Lidocaine‐releasing intravesical system (LiRIS) 400 mg G2 : placebo | 110 (G1 : 55, G2 : 55) | G1 : 48.6 (14.5), G2 : 44.2 (12.7) | 1 |
| FitzGerald et al 2009 | Western | RCT | patients with symptoms for less than 3 years. Patients must have previously undergone at least 1 course of another form of therapy for their symptoms | G1 : Myofascial Physical Therapy (MPT) G2 : Global Therapeutic Massage (GTM) | 26 (G1 : 12, G2 : 14) | G1 : 44.9 (14.0), G2 : 41.1 (11.4) | 3 |
| FitzGerald et al 2012 | Western | RCT | women with a clinical diagnosis of IC/PBS and recorded ratings for bladder pain, frequency and urgency, each at a usual level of at least 3 on a 0 to 10 scale, present for at least 3 months but not for longer than 3 years. | G1 : global therapeutic massage (GTM) - targeted internal and external tissue manipulation, focusing on the muscles and connective tissues of the pelvic floor, hip girdle and abdomen G2 : treatment with pelvic floor myofascial physical therapy (MPT) | 81 (G1 : 42, G2 : 39) | - | 3 |
| Kanter et al 2016 | Western | RCT | patients with IC/BPS. Negative urinalysis or urine culture within 2 months of enrolment and ability to speak and understand English. We recruited only patients who were currently undergoing first- or second-line treatments, as defined by the AUA (American Urological Association), and who had not made changes in their IC/BPS treatment regimen within 4 weeks of beginning their assigned intervention. | G1 : 8-week group mindfulness-based stress reduction meditation programme plus current care regimen. Also given 4-CD guide and a book G2 : usual care | 19 (G1 : 8, G2 : 11) | G1 : 46.3 (15.2), G2 : 44.4 (13.9) | 2 |
| Ko et al 2020 | Eastern | RCT | adults (aged 20 yr) to have bladder pain, urinary urgency, and frequency for at least 6 mo prior to study entry. patients must have had cystoscopic findings of HL within 2 yr of the study. | G1 : TUR was performed using a resectoscope with a bipolar loop electrode G2 : TUC was performed using resectoscope with a bipolar loop or rollerball | 122 (G1 : 63, G2 : 59) | G1 : 63 (56-70), G2 : 65 (56-71) | 1, 3, 6, 12 |
| Lee et al 2014 | Eastern | RCT | BPS/IC patients were recruited from the urological clinic of Taichung Hospital, Taichung, Taiwan | G1 : e-health system (Internet intervention to change habitual behaviour; no treatment, with questionnaires, SMS question/answer service for symptom relief) G2 : control | 80 (G1 : 40, G2 : 40) | G1 : 46.5 (10.2), G2 : 49.5 (11.8) | 2 |
| Lee et al 2018 | Eastern | RCT | IC/BPS patients were recruited from the urological clinic. If the patients met the inclusion and/or exclusion criterial of IC/BPS diagnosis and were willing to participate in the study | G1 : study group received video-based intervention accompanied with regular treatments G2 : control group received only regular treatments (outpatient clinics) | 56 (G1 : 29, G2 : 27) | G1 : 42.9 (10.4), G2 : 46.3 (14.2) | 2 |
| Matsumoto et al 2013 | Eastern | RCT | patients aged > 50 years with IC/PBS, who fulfilled the diagnostic criteria for IC proposed by the clinical guideline for IC. Stable history of IC/PBS symptoms ≥ 12 weeks after bladder hydrodistension, total ICSI score ≥ 7 and bladder pain (question 4 on ICSI) ≥ 4 | G1 : hydrogen-rich water 3 packs/d (1 pack, 200 mL), G2 : placebo water 3 packs/d (1 pack, 200 mL) | 28 (G1 : 18, G2 : 10) | G1 : 65.2 (7.9), G2 : 64.5 (4.5) | 2 |
| Oh-oka 2017 | Eastern | RCT | patients aged 20 years or older with diagnosis of IC/BPS. 1-year minimum duration of IC/BPS. The patient’s condition has been stable for at least 6 months after mild bladder hydrodistention. | G1 : intensive systematic dietary manipulation (ISDM) : information sharing (about foods to avoid) among physicians and other health-care professionals in the inpatient and outpatient settings, creation of a special menu, instruction to the cooking staff, monitoring in the kitchen, regular outpatient dietary counseling, and considerations of the burden placed on the staff associated with the administration of the ISDM program G2 : nonintensive DM (NIDM) | 40 (G1 : 30, G2 : 10) | G1 : 62.2 (0.4), G2 : 64.6 (1.5) | 3, 12 |
| O'Reilly et al 2004 | Western | RCT | patients with interstitial cystitis who fulfilled the National Institutes of Health National Institute for Diabetes and Digestive and Kidney Diseases criteria were prospectively recruited from our urogynaecology clinics. They were well-motivated patients in whom interstitial cystitis had failed to respond to various oral and intravesical therapies. | G1 : transdermal posterior tibial nerve stimulation using active device at home G2 : placebo device | 56 (G1 : 29, G2 : 27) | NA | 1, 3 |
| Peters et al 2007 | Western | RCT | Patients with refractory IC | An electrode was placed at the S3 nerve root and externalized. After placing a tined lead at S3, all patients had a second electrode placed on the same side at the pudendal nerve, via a posterior approach.  G1 : stimulation on the sacral electrode for 7 days G2 : stimulation on the pudendal electrode for 7 days | 17 (G1 : 4, G2 : 13) | 46 (26-70) | 6 |
| Soriano et al 2021 | Western | RCT | Included participants were at least 18 years old with a diagnosis of BPS/IC based on AUA criteria and score of ≥8 on the Interstitial Cystitis Symptom Index and Problem Index. | G1 : The hypnosis intervention included: (1) three standardized 18‐min one‐on‐one hypnosis sessions with a trained hypnotist over 4 weeks and (2) a web tool for daily home self‐hypnosis practice. The first and second sessions were 1 week apart while the second and third session were 2 weeks apart. G2 : usual care group was instructed to continue routine appointments and treatments with the provider managing their BPS/IC symptoms. | 29 (G1 : 15, G2 : 14) | G1 : 40.1 (17.0), G2 : 44.9 (20.0) | 1 |
| Van Ophoven et al 2006 | Western | RCT | patients 18 years of age or older who met diagnostic criteria of the NIDDK for IC | G1 : The Hyperbaric Oxygen (HBO) treatment schedule contained 30 treatment sessions of 100% oxygen inhalation via a facial mask at a chamber pressure of 2.4 ata in a multiplace hyperbaric chamber. Treatment was given in daily sessions, 6 times a week during a treatment period of 5 weeks.  G2 : The sham treatment was conducted identically to the described verum sessions except that the patients breathed normal air instead of 100% oxygen via a facial mask at a faintly increased pressure of 1.3 to 1.4 ata, to simulate compression and its effects on the tympanum. | 21 (G1 : 14, G2 : 7) | 65.5 (8.8) | 1, 3 |
| Geirsson et al 1993 | Western | RCT | female IC patients treated with various conservative measures with lack of response or recurrence of symptoms | G1 : acupuncture 2 to 3 times a week for 4 to 5 weeks G2 : transcutaneous tibial nerve stimulation (TENS) by patients at home, 30 minutes per day for 4 weeks | 12 (G1 : 6, G2 : 6) | median 41 (22-80) | 1 |

eTable 3. A reference list of included studies in the systematic review

| [1] Murina F, Graziottin A, Felice R, Gambini D. Alpha Lipoic Acid Plus Omega-3 Fatty Acids for Vestibulodynia Associated With Painful Bladder Syndrome. J Obstet Gynaecol Can. 2017;39:131-7. |
| --- |
| [2] Nickel JC, Barkin J, Forrest J, Mosbaugh PG, Hernandez-Graulau J, Kaufman D, et al. Randomized, double-blind, dose-ranging study of pentosan polysulfate sodium for interstitial cystitis. Urology. 2005;65:654-8. |
| [3] Nickel JC, Kaufman DM, Zhang HF, Wan GJ, Sand PK. Time to initiation of pentosan polysulfate sodium treatment after interstitial cystitis diagnosis: effect on symptom improvement. Urology. 2008;71:57-61. |
| [4] Sairanen J, Tammela TL, Leppilahti M, Multanen M, Paananen I, Lehtoranta K, et al. Cyclosporine A and pentosan polysulfate sodium for the treatment of interstitial cystitis: a randomized comparative study. J Urol. 2005;174:2235-8. |
| [5] Bosch PC. A randomized, double-blind, placebo controlled trial of adalimumab for interstitial cystitis/bladder pain syndrome. J Urol. 2014;191:77-82. |
| [6] Bosch PC. A Randomized, Double-blind, Placebo-controlled Trial of Certolizumab Pegol in Women with Refractory Interstitial Cystitis/Bladder Pain Syndrome. Eur Urol. 2018;74:623-30. |
| [7] Cartledge JJ, Davies AM, Eardley I. A randomized double-blind placebo-controlled crossover trial of the efficacy of L-arginine in the treatment of interstitial cystitis. BJU Int. 2000;85:421-6. |
| [8] Chen H, Wang F, Chen W, Ye X, Zhou Q, Shao F, et al. Efficacy of daily low-dose sildenafil for treating interstitial cystitis: results of a randomized, double-blind, placebo-controlled trial--treatment of interstitial cystitis/painful bladder syndrome with low-dose sildenafil. Urology. 2014;84:51-6. |
| [9] Evans RJ, Moldwin RM, Cossons N, Darekar A, Mills IW, Scholfield D. Proof of concept trial of tanezumab for the treatment of symptoms associated with interstitial cystitis. J Urol. 2011;185:1716-21. |
| [10] Foster HE, Jr., Hanno PM, Nickel JC, Payne CK, Mayer RD, Burks DA, et al. Effect of amitriptyline on symptoms in treatment naïve patients with interstitial cystitis/painful bladder syndrome. J Urol. 2010;183:1853-8. |
| [11] Houbiers JGA, van Till JWO, Kaper M, Yavuz Y, Martina RV, Cerneus D, et al. An adaptive randomized clinical trial in interstitial cystitis/bladder pain syndrome evaluating efficacy of ASP3652 and the relationship between disease characteristics and Hunner's lesions. World J Urol. 2021;39:2065-71. |
| [12] Kasyan G, Kupriyanov Y, Karasev A, Baibarin K, Pushkar D. Safety and efficacy of pentosan polysulfate in patients with bladder pain syndrome/interstitial cystitis: A multicenter, double–blind, placebo–controlled, randomized study. Central European Journal of Urology. 2021;74:201-7. |
| [13] Korting GE, Smith SD, Wheeler MA, Weiss RM, Foster HE, Jr. A randomized double-blind trial of oral L-arginine for treatment of interstitial cystitis. J Urol. 1999;161:558-65. |
| [14] Lazzeri M, Beneforti P, Spinelli M, Zanollo A, Barbagli G, Turini D. Intravesical resiniferatoxin for the treatment of hypersensitive disorder: A randomized placebo controlled study. Journal of Urology. 2000;164:676-9. |
| [15] Nickel JC, Egerdie B, Davis E, Evans R, Mackenzie L, Shrewsbury SB. A Phase II Study of the Efficacy and Safety of the Novel Oral SHIP1 Activator AQX-1125 in Subjects with Moderate to Severe Interstitial Cystitis/Bladder Pain Syndrome. J Urol. 2016;196:747-54. |
| [16] Nickel JC, Herschorn S, Whitmore KE, Forrest JB, Hu P, Friedman AJ, et al. Pentosan polysulfate sodium for treatment of interstitial cystitis/bladder pain syndrome: insights from a randomized, double-blind, placebo controlled study. J Urol. 2015;193:857-62. |
| [17] Nickel JC, Moldwin R, Hanno P, Dmochowski R, Peters KM, Payne C, et al. Targeting the SHIP1 Pathway Fails to Show Treatment Benefit in Interstitial Cystitis/Bladder Pain Syndrome: Lessons Learned from Evaluating Potentially Effective Therapies in This Enigmatic Syndrome. J Urol. 2019;202:301-8. |
| [18] Sant GR, Propert KJ, Hanno PM, Burks D, Culkin D, Diokno AC, et al. A pilot clinical trial of oral pentosan polysulfate and oral hydroxyzine in patients with interstitial cystitis. Journal of Urology. 2003;170:810-5. |
| [19] Souza GHB, Maistro EL, Rodrigues M, Carvalho JCT, Fonseca FLA, Lopes AP, et al. Phase II clinical study of an association for the treatment of interstitial cystitis (Cystex®). HealthMED. 2012;6:423-7. |
| [20] van Ophoven A, Pokupic S, Heinecke A, Hertle L. A prospective, randomized, placebo controlled, double-blind study of amitriptyline for the treatment of interstitial cystitis. J Urol. 2004;172:533-6. |
| [21] Wang H, Russell LJ, Kelly KM, Wang S, Thipphawong J. Fulranumab in patients with interstitial cystitis/bladder pain syndrome: observations from a randomized, double-blind, placebo-controlled study. BMC Urol. 2017;17:2. |
| [22] Yang CC, Burks DA, Propert KJ, Mayer RD, Peters KM, Nickel JC, et al. Early termination of a trial of mycophenolate mofetil for treatment of interstitial cystitis/painful bladder syndrome: lessons learned. J Urol. 2011;185:901-6. |
| [23] Banakhar MA. Comparative effectiveness and safety of cocktail therapy versus combined sodium hyaluronate and chondroitin sulphate (Ialuril): Intravesical instillation treatment of interstitial cystitis/bladder pain syndrome, which one to use? Journal of Clinical Urology. 2022. |
| [24] Cardenas-Trowers O, Goodman-Abraham A, Dotson T, Houlette B, Gaskins J, Francis S. Effect of bladder instillations with versus without triamcinolone acetonide on symptoms of interstitial cystitis/bladder pain syndrome in women: A randomized controlled trial. Neurourology and Urodynamics. 2021;40:S39-S40. |
| [25] Cervigni M, Sommariva M, Tenaglia R, Porru D, Ostardo E, Giammò A, et al. A randomized, open-label, multicenter study of the efficacy and safety of intravesical hyaluronic acid and chondroitin sulfate versus dimethyl sulfoxide in women with bladder pain syndrome/interstitial cystitis. Neurourol Urodyn. 2017;36:1178-86. |
| [26] El-Bahnasy AE. A randomized controlled trial of bacillus Calmette-Guerin and botulinum toxin-A for the treatment of refractory interstitial cystitis. Uro Today Int J. 2009. |
| [27] Gülpinar O, Haliloǧlu AH, Gökce MI, Arikan N. Instillation of hyaluronic acid via electromotive drug administration can improve the efficacy of treatment in patients with interstitial cystitis/painful bladder syndrome: A randomized prospective study. Korean Journal of Urology. 2014;55:354-9. |
| [28] Gülpınar Ö, Esen B, Kayış A, Gökçe M, Süer E. Clinical comparison of intravesical hyaluronic acid and chondroitin sulfate therapies in the treatment of bladder pain syndrome/interstitial cystitis. Neurourol Urodyn. 2018;37:257-62. |
| [29] Guo-liang L, Da-wei W, Yuan S. Comparative study on intravesical hyaluronic acid instillation and heparin-lidocaine instillation for the treatment of interstitial cystitis. Journal of Shanghai Jiaotong University (Medical Science). 2015;35:1757. |
| [30] Lai MC, Kuo YC, Kuo HC. Intravesical hyaluronic acid for interstitial cystitis/painful bladder syndrome: a comparative randomized assessment of different regimens. Int J Urol. 2013;20:203-7. |
| [31] Özkidik M. Assessment of long-term intravesical hyaluronic acid, chondroitin sulfate and combination therapy for patients with bladder pain syndrome. Central European Journal of Urology. 2019;72:270-5. |
| [32] Peeker R, Haghsheno MA, Holmäng S, Fall M. Intravesical bacillus Calmette-Guerin and dimethyl sulfoxide for treatment of classic and nonulcer interstitial cystitis: a prospective, randomized double-blind study. J Urol. 2000;164:1912-5; discussion 5-6. |
| [33] Barbalias GA, Liatsikos EN, Athanasopoulos A, Nikiforidis G. Interstitial cystitis: bladder training with intravesical oxybutynin. J Urol. 2000;163:1818-22. |
| [34] Chen TY, Corcos J, Camel M, Ponsot Y, Tu le M. Prospective, randomized, double-blind study of safety and tolerability of intravesical resiniferatoxin (RTX) in interstitial cystitis (IC). Int Urogynecol J Pelvic Floor Dysfunct. 2005;16:293-7. |
| [35] Chuang YC, Kuo HC. A Prospective, Multicenter, Double-Blind, Randomized Trial of Bladder Instillation of Liposome Formulation OnabotulinumtoxinA for Interstitial Cystitis/Bladder Pain Syndrome. J Urol. 2017;198:376-82. |
| [36] Cvach K, Rosamilia A, Dwyer P, Lim Y, DeSouza A, Ow L, et al. Efficacy of Clorpactin in refractory bladder pain syndrome/interstitial cystitis: a randomized controlled trial. Int Urogynecol J. 2021;32:1177-83. |
| [37] Davis EL, El Khoudary SR, Talbott EO, Davis J, Regan LJ. Safety and efficacy of the use of intravesical and oral pentosan polysulfate sodium for interstitial cystitis: a randomized double-blind clinical trial. J Urol. 2008;179:177-85. |
| [38] Ham BK, Kim JH, Oh MM, Lee JG, Bae JH. Effects of combination treatment of intravesical resiniferatoxin instillation and hydrodistention in patients with refractory painful bladder syndrome/interstitial cystitis: a pilot study. Int Neurourol J. 2012;16:41-6. |
| [39] Irani D, Heidari M, Khezri AA. The efficacy and safety of intravesical Bacillus-Calmette-Guerin in the treatment of female patients with interstitial cystitis: a double-blinded prospective placebo controlled study. Urol J. 2004;1:90-3. |
| [40] Mayer R, Propert KJ, Peters KM, Payne CK, Zhang Y, Burks D, et al. A randomized controlled trial of intravesical bacillus calmette-guerin for treatment refractory interstitial cystitis. J Urol. 2005;173:1186-91. |
| [41] Nickel JC, Moldwin R, Lee S, Davis EL, Henry RA, Wyllie MG. Intravesical alkalinized lidocaine (PSD597) offers sustained relief from symptoms of interstitial cystitis and painful bladder syndrome. BJU Int. 2009;103:910-8. |
| [42] Nickel JC, Egerdie RB, Steinhoff G, Palmer B, Hanno P. A multicenter, randomized, double-blind, parallel group pilot evaluation of the efficacy and safety of intravesical sodium chondroitin sulfate versus vehicle control in patients with interstitial cystitis/painful bladder syndrome. Urology. 2010;76:804-9. |
| [43] Nickel JC, Hanno P, Kumar K, Thomas H. Second multicenter, randomized, double-blind, parallel-group evaluation of effectiveness and safety of intravesical sodium chondroitin sulfate compared with inactive vehicle control in subjects with interstitial cystitis/bladder pain syndrome. Urology. 2012;79:1220-4. |
| [44] Propert KJ, Mayer R, Nickel JC, Payne CK, Peters KM, Teal V, et al. Followup of patients with interstitial cystitis responsive to treatment with intravesical bacillus Calmette-Guerin or placebo. J Urol. 2008;179:552-5. |
| [45] Yoshimura N, Homma Y, Tomoe H, Otsuka A, Kitta T, Masumori N, et al. Efficacy and safety of intravesical instillation of KRP-116D (50% dimethyl sulfoxide solution) for interstitial cystitis/bladder pain syndrome in Japanese patients: A multicenter, randomized, double-blind, placebo-controlled, clinical study. Int J Urol. 2021;28:545-53. |
| [46] Hsieh CH, Chang WC, Huang MC, Su TH, Li YT, Chang ST, et al. Hydrodistention plus bladder training versus hydrodistention for the treatment of interstitial cystitis. Taiwan J Obstet Gynecol. 2012;51:591-5. |
| [47] Akiyama Y, Nomiya A, Niimi A, Yamada Y, Fujimura T, Nakagawa T, et al. Botulinum toxin type A injection for refractory interstitial cystitis: A randomized comparative study and predictors of treatment response. Int J Urol. 2015;22:835-41. |
| [48] Jiang YH, Jhang JF, Lee CL, Kuo HC. Comparative study of efficacy and safety between bladder body and trigonal intravesical onabotulinumtoxina injection in the treatment of interstitial cystitis refractory to conventional treatment-A prospective, randomized, clinical trial. Neurourol Urodyn. 2018;37:1467-73. |
| [49] Kuo HC, Chancellor MB. Comparison of intravesical botulinum toxin type A injections plus hydrodistention with hydrodistention alone for the treatment of refractory interstitial cystitis/painful bladder syndrome. BJU Int. 2009;104:657-61. |
| [50] Kuo HC, Jiang YH, Tsai YC, Kuo YC. Intravesical botulinum toxin-A injections reduce bladder pain of interstitial cystitis/bladder pain syndrome refractory to conventional treatment - A prospective, multicenter, randomized, double-blind, placebo-controlled clinical trial. Neurourol Urodyn. 2016;35:609-14. |
| [51] Manning J, Dwyer P, Rosamilia A, Colyvas K, Murray C, Fitzgerald E. A multicentre, prospective, randomised, double-blind study to measure the treatment effectiveness of abobotulinum A (AboBTXA) among women with refractory interstitial cystitis/bladder pain syndrome. Int Urogynecol J. 2014;25:593-9. |
| [52] Payne CK, Mosbaugh PG, Forrest JB, Evans RJ, Whitmore KE, Antoci JP, et al. Intravesical resiniferatoxin for the treatment of interstitial cystitis: a randomized, double-blind, placebo controlled trial. J Urol. 2005;173:1590-4. |
| [53] Pinto RA, Costa D, Morgado A, Pereira P, Charrua A, Silva J, et al. Intratrigonal OnabotulinumtoxinA Improves Bladder Symptoms and Quality of Life in Patients with Bladder Pain Syndrome/Interstitial Cystitis: A Pilot, Single Center, Randomized, Double-Blind, Placebo Controlled Trial. J Urol. 2018;199:998-1003. |
| [54] Carralero-Martínez A, Muñoz Pérez MA, Kauffmann S, Blanco-Ratto L, Ramírez-García I. Efficacy of capacitive resistive monopolar radiofrequency in the physiotherapeutic treatment of chronic pelvic pain syndrome: A randomized controlled trial. Neurourology and Urodynamics. 2022. |
| [55] Chuang YC, Meng E, Chancellor M, Kuo HC. Pain reduction realized with extracorporeal shock wave therapy for the treatment of symptoms associated with interstitial cystitis/bladder pain syndrome-A prospective, multicenter, randomized, double-blind, placebo-controlled study. Neurourol Urodyn. 2020;39:1505-14. |
| [56] El-Hefnawy AS, Makharita MY, Abed A, Amr YM, Salah El-Badry M, Shaaban AA. Anesthetic Bladder Hydrodistention Is Superior to Superior Hypogastric Plexus Neurolysis in Treatment of Interstitial Cystitis-bladder Pain Syndrome: A Prospective Randomized Trial. Urology. 2015;85:1039-44. |
| [57] Evans R, Kohan A, Moldwin R, Radecki D, Geib T, Peters KM. Safety, tolerability, and efficacy of LiRIS 400 mg in women with interstitial cystitis/bladder pain syndrome with or without Hunner lesions. Neurourol Urodyn. 2021;40:1730-9. |
| [58] FitzGerald MP, Anderson RU, Potts J, Payne CK, Peters KM, Clemens JQ, et al. Randomized Multicenter Feasibility Trial of Myofascial Physical Therapy for the Treatment of Urological Chronic Pelvic Pain Syndromes. Journal of Urology. 2009;182:570-80. |
| [59] FitzGerald MP, Payne CK, Lukacz ES, Yang CC, Peters KM, Chai TC, et al. Randomized multicenter clinical trial of myofascial physical therapy in women with interstitial cystitis/painful bladder syndrome and pelvic floor tenderness. J Urol. 2012;187:2113-8. |
| [60] Kanter G, Komesu YM, Qaedan F, Jeppson PC, Dunivan GC, Cichowski SB, et al. Mindfulness-based stress reduction as a novel treatment for interstitial cystitis/bladder pain syndrome: a randomized controlled trial. Int Urogynecol J. 2016;27:1705-11. |
| [61] Ko KJ, Cho WJ, Lee YS, Choi J, Byun HJ, Lee KS. Comparison of the Efficacy Between Transurethral Coagulation and Transurethral Resection of Hunner Lesion in Interstitial Cystitis/Bladder Pain Syndrome Patients: A Prospective Randomized Controlled Trial. Eur Urol. 2020;77:644-51. |
| [62] Lee MH, Wu HC, Lin JY, Tan TH, Chan PC, Chen YF. Development and evaluation of an E-health system to care for patients with bladder pain syndrome/interstitial cystitis. Int J Urol. 2014;21 Suppl 1:62-8. |
| [63] Lee MH, Wu HC, Tseng CM, Ko TL, Weng TJ, Chen YF. Health Education and Symptom Flare Management Using a Video-based m-Health System for Caring Women With IC/BPS. Urology. 2018;119:62-9. |
| [64]] Matsumoto S, Ueda T, Kakizaki H. Effect of supplementation with hydrogen-rich water in patients with interstitial cystitis/painful bladder syndrome. Urology. 2013;81:226-30. |
| [65]] Oh-Oka H. Clinical Efficacy of 1-Year Intensive Systematic Dietary Manipulation as Complementary and Alternative Medicine Therapies on Female Patients With Interstitial Cystitis/Bladder Pain Syndrome. Urology. 2017;106:50-4. |
| [66] O'Reilly BA, Dwyer PL, Hawthorne G, Cleaver S, Thomas E, Rosamilia A, et al. Transdermal posterior tibial nerve laser therapy is not effective in women with interstitial cystitis. J Urol. 2004;172:1880-3. |
| [67] Peters KM, Feber KM, Bennett RC. A prospective, single-blind, randomized crossover trial of sacral vs pudendal nerve stimulation for interstitial cystitis. BJU Int. 2007;100:835-9. |
| [68] Soriano AJ, Schnur JB, Harvie HS, Newman DK, Montgomery GH, Arya LA. Pilot randomized controlled trial of a hypnosis intervention for women with bladder pain syndrome. Neurourol Urodyn. 2021;40:1945-54. |
| [69] van Ophoven A, Rossbach G, Pajonk F, Hertle L. Safety and efficacy of hyperbaric oxygen therapy for the treatment of interstitial cystitis: a randomized, sham controlled, double-blind trial. J Urol. 2006;176:1442-6. |
| [70] Geirsson G, Wang YH, Lindström S, Fall M. Traditional acupuncture and electrical stimulation of the posterior tibial nerve. A trial in chronic interstitial cystitis. Scand J Urol Nephrol. 1993;27:67-70. |

eTable 4. Specific treatments and outcomes in the meta-analysis

|  | **Study** | **Treatment class** | **Treatments** | **outcomes** |
| --- | --- | --- | --- | --- |
| **Medication or system injection** | |  |  |  |
|  | Murina et al. 2017 | antidepressants | - amitriptyline | VAS pain score |
|  |  | antidepressants plus nutritional supplements | - amitriptyline plus a commercially available preparation containing ALA 600 mg, DHA 250 mg, and eicosapentaenoic acid 16.67 mg | VAS pain score |
|  | Sairanen et al. 2005 | old immune modulator | - cyclosporine (3 mg/kg divided into 2 daily) | VAS pain score, ICSI, ICPI |
|  |  | PPS | - PPS (100 mg 3 times daily) | VAS pain score, ICSI, ICPI |
|  | Bosch et al. 2014 | new immune modulator | - 80 mg loading dose of subcutaneous adalimumab followed by 40 mg every 2 weeks | ICSI, ICPI, PUF |
|  |  | control | - subcutaneous placebo | ICSI, ICPI, PUF |
|  | Bosch et al. 2018 | new immune modulator | - subcutaneous certolizumab pegol 400 mg at 0, 2, 4, 8 weeks | ICSI, ICPI, 11-point numerical rating scale (pain, urgency) |
|  |  | control | - subcutaneous placebo (sterile saline) at 0, 2, 4, 8 weeks | ICSI, ICPI, 11-point numerical rating scale (pain, urgency) |
|  | Cartledge et al. 2000 | amino acid | - L-arginine (2.4 g/day) for one month | ICSI |
|  |  | control | - placebo for one month | ICSI |
|  | Foster et al. 2010 | antidepressants | - Amitriptyline (dose was increased on a weekly basis from 10mg to 75mg, then maintained at the highest tolerable dose until 12 weeks) with standardized education and behavioral modification program | ICSI, ICPI, 11-point numerical rating scale (pain, urgency) |
|  |  | control | - placebo with standardized education and behavioral modification program | ICSI, ICPI, 11-point numerical rating scale (pain, urgency) |
|  | Houbiers et al. 2021 | ASP3652 100mg | - ASP3652 50mg bid | ICSI, ICPI, frequency per 24h, urgency episodes per 24h |
|  |  | ASP3652 300mg | - ASP3652 150mg bid | ICSI, ICPI, frequency per 24h, urgency episodes per 24h |
|  |  | ASP3652 600mg | - ASP3652 300mg bid | ICSI, ICPI, frequency per 24h, urgency episodes per 24h |
|  |  | control | - placebo | ICSI, ICPI, frequency per 24h, urgency episodes per 24h |
|  | Kasyan et al. 2021 | PPS | - Pentosane polysulfate 100 mg tid | ICSI, ICPI |
|  |  | control | - placebo 100 mg tid | ICSI, ICPI |
|  | Korting et al. 1999 | amino acid | - L-arginine 500 mg tid with usual care for 3 months | frequency per 24h, nocturia per day |
|  |  | control | - placebo tid with usual care for 3 months | frequency per 24h, nocturia per day |
|  | Lazzeri et al 2000 | RTX | - resiniferatoxin (RTX) 10 im in 0.1% ethanol | VAS pain score, frequency per 24h, nocturia per day |
|  |  | control | - NaCl | VAS pain score, frequency per 24h, nocturia per day |
|  | Nickel et al. 2015 | PPS | - PPS 100 mg QD | 11-point numerical rating scale (pain), frequency per 24h |
|  |  | control | - placebo | 11-point numerical rating scale (pain), frequency per 24h |
|  | Sant et al. 2003 | PPS | - PPS (100 mg tid) | ICSI, ICPI, frequency per 24h |
|  |  | antihistamine | - Hydroxyzine (10-50mg daily) | ICSI, ICPI, frequency per 24h |
|  |  | PPS plus antihistamine | - combination therapy (PPS (100 mg tid) + Hydroxyzine (10-50mg daily)) | ICSI, ICPI, frequency per 24h |
|  |  | control | - Placebo | ICSI, ICPI, frequency per 24h |
|  | Souza et al. 2012 | analgesics | - Cystex® capsule (Acriflavin hydrochloride 15.00 mg; Methenamine 250.00 mg; Methylene blue 20.00 mg; Beladona extract 15.00 mg) for 21 days | VAS pain score, VAS urgency score |
|  |  | control | - placebo for 21 days | VAS pain score, VAS urgency score |
|  | Van Ophoven et al. 2004 | antidepressants | - amitriptyline 25-100mg at bedtime (maximum allowed dosage) | VAS pain score, VAS urgency score, frequency per 24h, functional bladder volume (ml) |
|  |  | control | - placebo | VAS pain score, VAS urgency score, frequency per 24h, functional bladder volume (ml) |
|  | Wang et al. 2017 | new immune modulator | - fulranumab 9 mg injected subcutaneously (SC) into the thigh once every 4 weeks | ICSI, PUF, 11-point numerical rating scale (pain), frequency per 24h, nocturia per day |
|  |  | control | - placebo injected subcutaneously (SC) into the thigh once every 4 weeks | ICSI, PUF, 11-point numerical rating scale (pain), frequency per 24h, nocturia per day |
|  | Yang et al 2011 | old immune modulator | - immunosuppressant 1 gram daily, mycophenolate mofetil (MMF) for 14 days, then 1 gram BID for 10 weeks | VAS pain score, ICSI, ICPI |
|  |  | control | - placebo | VAS pain score, ICSI, ICPI |
| **Instillation** | |  |  |  |
|  | Banakhar 2022 | coktail | - cocktail intravesical therapy (NaOH + heparin + lidocaine) | ICSI, ICPI |
|  |  | HA plus CS | - Ialuril (HA and CS) | ICSI, ICPI |
|  | Cardenas-Trowers et al. 2021 | immune modulators | - 6 bladder instillations with triamcinolone acetonide | VAS urgency score, ICSI, ICPI |
|  |  | control | - 6 bladder instillations without triamcinolone acetonide | VAS urgency score, ICSI, ICPI |
|  | Cervigni et al. 2017 | HA plus CS | - 13 weekly instillations of HA (1.6%) and CS (2.0%) (Ialuril®; IBSA) | VAS urgency score, ICSI, ICPI, frequency per 24h, functional bladder volume (ml) |
|  |  | DMSO | - 13 weekly instillations of 50% DMSO solution (RIMSO®; Bioniche) | VAS urgency score, ICSI, ICPI, frequency per 24h, functional bladder volume (ml) |
|  | El-Bahnasy et al 2008 | BCG | - intravesical BCG administration in the form of weekly instillations | VAS pain score, frequency per 24h, nocturia per day, Urgency episodes per 24h, nocturia per day |
|  |  | BTX | - intravesical injections of BTX-A under local anesthesia in the form of intraurethral 2% lidocaine jell and intravesical 2% lidocaine on 100 ml saline for 10 minutes | VAS pain score, frequency per 24h, nocturia per day, Urgency episodes per 24h, nocturia per day |
|  | Gülpınar et al. 2014 | HA | - 40mg hyaluronic acid intravesical instillation and retaining for at least 60 mins | VAS pain score, ICSI, ICPI, frequency per 24h, functional bladder volume (ml) |
|  |  | HA plus electric device | - 40mg hyaluronic acid and electromotive drug administration (EMDA) intravesical instillation and retaining for at least 60 mins. (using the generator) | VAS pain score, ICSI, ICPI, frequency per 24h, functional bladder volume (ml) |
|  | Gülpınar et al. 2018 | HA | - intravesical therapy with 50 mL/120 mg sterile sodium hyaluronic acid (HA) | frequency per 24h |
|  |  | CS | - intravesical therapy with 40 mL/80 mg sodium chondroitin suphate (CS) | frequency per 24h |
|  | Guo-liang et al 2015 | HA | - intravesical hyaluronic acid (HA) after bladder hydrodistention | VAS pain score, frequency per 24h |
|  |  | lidocaine | - intravesical heparin sodium-lidocaine (HL) after bladder hydrodistention | VAS pain score, frequency per 24h |
|  | Ozkıdık et al. 2019 | HA | - intravesical instillation of hyaluronic acid (HA) | VAS pain score, ICSI, ICPI, frequency per 24h, functional bladder volume (ml), Urgency episodes per 24h |
|  |  | CS | - intravesical instillation of chondroitin sulphate (CS) instillation | VAS pain score, ICSI, ICPI, frequency per 24h, functional bladder volume (ml), Urgency episodes per 24h |
|  |  | HA plus CS | - intravesical instillation of combination of HA and CS instillation | VAS pain score, ICSI, ICPI, frequency per 24h, functional bladder volume (ml), Urgency episodes per 24h |
|  | Chuang et al. 2017 | BTX plus sphingomyelin | - intravesical instillation of lipotoxin (onabotulinumtoxinA 200 U with 80 mg sphingomyelin) | VAS pain score, ICSI, ICPI, frequency per 24h, nocturia per day, functional bladder volume (ml) |
|  |  | BTX | - intravesical instillation of onabotulinumtoxinA 200 U in normal saline | VAS pain score, ICSI, ICPI, frequency per 24h, nocturia per day, functional bladder volume (ml) |
|  |  | control | - intravesical instillation of normal saline alone | VAS pain score, ICSI, ICPI, frequency per 24h, nocturia per day, functional bladder volume (ml) |
|  | Davis et al 2008 | PPS | - Intravesical PPS (twice a week) during the first six weeks + oral PPS (200mg/twice a day) during the entire period of the study | VAS pain score, ICSI, ICPI, frequency per 24h, nocturia per day |
|  |  | control | - Intravesical placebo during the first six weeks + orak PPS (200mg / twice a day) during the entire period of the study | VAS pain score, ICSI, ICPI, frequency per 24h, nocturia per day |
|  | Irani et al 2004 | BCG | - intravesical installation of 120 mg of BCG vaccine through foley catheter for six weeks | VAS pain score, frequency per 24h, nocturia per day, functional bladder volume (ml) |
|  |  | control | - intravesical installation of 50 cc of normal saline (as placebo) through foley catheter for six weeks | VAS pain score, frequency per 24h, nocturia per day, functional bladder volume (ml) |
|  | Mayer et al. 2005 | BCG | - intravesical instillation of BCG up to 6 times within 6 to 10 weeks | VAS pain score, ICSI, ICPI, frequency per 24h, functional bladder volume (ml) |
|  |  | control | - intravesical instillation of placebo up to 6 times within 6 to 10 weeks | VAS pain score, ICSI, ICPI, frequency per 24h, functional bladder volume (ml) |
|  | Nickel et al. 2008 | lidocaine | - intravesical instillation of PSD597 (a patented combination of 200 mg lidocaine, alkalinized with a sequential instillation of 8.4% sodium bicarbonate solution, to a final volume of 10 mL) once a day for 5 consecutive days | ICSI, ICPI, frequency per 24h |
|  |  | control | - intravesical instillation of placebo (10 mL of ordinary saline) once a day for 5 consecutive days | ICSI, ICPI, frequency per 24h |
|  | Nickel et al. 2010 | CS | - intravesical instillation of 20 mL of a sterile 2.0% solution of sodium chondroitin sulfate (Uracyst, Watson Pharmaceuticals, Corona, CA) weekly for 6 weeks | ICSI, ICPI, frequency per 24h |
|  |  | control | - intravesical instillation of 20 mL sterile vehicle control solution (the identical phosphate-buffered saline vehicle used for Uracyst) weekly for 6 weeks | ICSI, ICPI, frequency per 24h |
|  | Nickel et al. 2012 | CS | - intravesical instillation of 20 mL of 2% chondroitin sulfate once weekly for 7 weeks (8 treatments in total) | VAS pain score, ICSI, ICPI, frequency per 24h, Urgency episodes per 24h, functional bladder volume (ml) |
|  |  | control | - intravesical instillation of 20 mL of inactive control (the identical phosphate-buffered saline vehicle used for Uracyst) once weekly for 7 weeks | VAS pain score, ICSI, ICPI, frequency per 24h, Urgency episodes per 24h, functional bladder volume (ml) |
|  | Propert et al. 2008 | BCG | - up to six intravesical instillations of BCG treatment, over a six to ten week period | ICSI, ICPI, frequency per 24h, Urgency episodes per 24h |
|  |  | control | - up to six intravesical instillations of saline (placebo) treatment, over a six to ten week period | ICSI, ICPI, frequency per 24h, Urgency episodes per 24h |
|  | Yoshimura et al. 2021 | DMSO | - intravesical instillation of KRP-116D (50% DMSO solution) every 2 weeks for 12 weeks | ICSI, ICPI, 11-point numerical rating scale (pain), frequency per 24h, functional bladder volume (ml) |
|  |  | control | - intravesical instillation of placebo every 2 weeks for 12 weeks | ICSI, ICPI, 11-point numerical rating scale (pain), frequency per 24h, functional bladder volume (ml) |
| **Intravesical injection** | |  |  |  |
|  | Kuo et al 2009 | BTX 200U | - intravesical injection of 200 U of BoNT-A (BOTOX, Allergan, Irvine, CA, USA) | VAS pain score, ICSI, ICPI, frequency per 24h, nocturia per day, functional bladder volume (ml) |
|  |  | BTX 100U | - intravesical injection of 100 U of BoNT-A | VAS pain score, ICSI, ICPI, frequency per 24h, nocturia per day, functional bladder volume (ml) |
|  |  | control | - cystoscopic HD alone | VAS pain score, ICSI, ICPI, frequency per 24h, nocturia per day, functional bladder volume (ml) |
|  | Kuo et al 2016 | BTX 100U | - intravesical injection of 100U of BoNT-A (BOTOX, Allergan, Irvine,CA) immediately followed by cystoscopic hydrodistention | VAS pain score, ICSI, ICPI, frequency per 24h, nocturia per day, functional bladder volume (ml) |
|  |  | control | - intravesical injection with normal saline immediately followed by cystoscopic hydrodistention | VAS pain score, ICSI, ICPI, frequency per 24h, nocturia per day, functional bladder volume (ml) |
|  | Payne et al 2005 | RTX 0.01 μM | - single intravesical dose of RTX 0.01 μM | VAS pain score |
|  |  | RTX 0.05 μM | - single intravesical dose of RTX 0.05 μM | VAS pain score |
|  |  | RTX 0.1 μM | - single intravesical dose of RTX 0.1 μM | VAS pain score |
|  |  | control | - single intravesical dose of placebo | VAS pain score |
|  | Pinto et al 2018 | BTX 100U | - 10 injections of 1 ml 0.9% sodium chloride containing 10 U OnaBotA (total 10 ml and 100 U, respectively) | VAS pain score, ICSI, frequency per 24h |
|  |  | control | - 10 injections of 1 ml 0.9% sodium chloride | VAS pain score, ICSI, frequency per 24h |
|  | Evans et al 2021 | anesthetics | - a single treatment of Lidocaine‐releasing intravesical system (LiRIS) 400 mg | VAS pain score, frequency per 24h |
|  |  | control | - placebo | VAS pain score, frequency per 24h |
| **Physical therapy** | |  |  |  |
|  | Carralero-Martinez et al 2022 | radiofrequency | - Treatment consisted of 10 capacitive resistive monopolar radiofrequency (CRMRF) sessions | VAS pain score |
|  |  | control | - Treatment consisted of 10 unactivated CRMRF sessions | VAS pain score |
|  | O'Reilly et al 2004 | nerve stimulation | - transdermal posterior tibial nerve stimulation using active device at home | ICSI, ICPI, frequency per 24h |
|  |  | control | - placebo device | ICSI, ICPI, frequency per 24h |
| **Others** | |  |  |  |
|  | Kanter et al 2016 | meditation | - 8-week group mindfulness-based stress reduction meditation programme plus current care regimen. Also given 4-CD guide and a book | VAS urgency score, ICSI, ICPI |
|  |  | control | - usual care | VAS urgency score, ICSI, ICPI |
|  | Lee et al 2014 | meditation | - e-health system (Internet intervention to change habitual behaviour; no treatment, with questionnaires, SMS question/answer service for symptom relief) | VAS pain score, VAS urgency score, ICSI, ICPI |
|  |  | control | - Control group | VAS pain score, VAS urgency score, ICSI, ICPI |
|  | Lee et al 2018 | video intervention | - study group received video-based intervention accompanied with regular treatments | VAS pain score, VAS urgency score, ICSI, ICPI |
|  |  | control | - control group received only regular treatments (outpatient clinics) | VAS pain score, VAS urgency score, ICSI, ICPI |
|  | Matsumoto et al 2013 | hydrogen rich water | - hydrogen-rich water 3 packs/d (1 pack, 200 mL) | VAS pain score, ICSI, ICPI, frequency per 24h, functional bladder volume (ml) |
|  |  | control | - placebo water 3 packs/d (1 pack, 200 mL) | VAS pain score, ICSI, ICPI, frequency per 24h, functional bladder volume (ml) |
|  | Oh-oka 2017 | dietary | - intensive systematic dietary manipulation (ISDM) : information sharing (about foods to avoid) among physicians and other health-care professionals in the inpatient and outpatient settings, creation of a special menu, instruction to the cooking staff, monitoring in the kitchen, regular outpatient dietary counseling, and considerations of the burden placed on the staff associated with the administration of the ISDM program | VAS pain score, ICSI, ICPI, urgency episodes per 24h |
|  |  | control | - nonintensive DM (NIDM) | VAS pain score, ICSI, ICPI, urgency episodes per 24h |
|  | Soriano et al 2021 | hypnosis | - The hypnosis intervention included: (1) three standardized 18‐min one‐on‐one hypnosis sessions with a trained hypnotist over 4 weeks and (2) a web tool for daily home self‐hypnosis practice. The first and second sessions were 1 week apart while the second and third session were 2 weeks apart. | ICSI, ICPI |
|  |  | control | - usual care group was instructed to continue routine appointments and treatments with the provider managing their BPS/IC symptoms. | ICSI, ICPI |
|  | Van Ophoven et al 2006 | hyperharic oxygen | - The Hyperbaric Oxygen (HBO) treatment schedule contained 30 treatment sessions of 100% oxygen inhalation via a facial mask at a chamber pressure of 2.4 ata in a multiplace hyperbaric chamber. Treatment was given in daily sessions, 6 times a week during a treatment period of 5 weeks. | VAS pain score, VAS urgency score, ICSI, ICPI, frequency per 24h, functional bladder volume (ml) |
|  |  | control | - The sham treatment was conducted identically to the described verum sessions except that the patients breathed normal air instead of 100% oxygen via a facial mask at a faintly increased pressure of 1.3 to 1.4 ata, to simulate compression and its effects on the tympanum. | VAS pain score, VAS urgency score, ICSI, ICPI, frequency per 24h, functional bladder volume (ml) |

eTable 5. Moderator effects of structured questionnaires for interstitial cystitis in pairwise meta-analysis

|  | **ICPI** | | | | | |  | **ICSI** | | | | | |  | **PUF** | | | | | |  | **FBV** | | | | | |
| --- | --- | --- | --- | --- | --- | --- | --- | --- | --- | --- | --- | --- | --- | --- | --- | --- | --- | --- | --- | --- | --- | --- | --- | --- | --- | --- | --- |
| Variables | *k* | *β* | SMD | 95% CI | | *p* |  | *k* | *β* | SMD | 95% CI | | *p* |  | *k* | *β* | SMD | 95% CI | | *p* |  | *k* | *β* | SMD | 95% CI | | *p* |
| No. of total patients | 28 | 0.001 |  | -0.003 | 0.006 | 0.521 |  | 31 | 0.000 |  | -0.003 | 0.004 | 0.953 |  | 4 | -0.017 |  | -0.041 | 0.006 | 0.148 |  | 12 | -0.003 |  | -0.006 | 0.000 | 0.061 |
| Age | 25 | 0.010 |  | -0.015 | 0.035 | 0.444 |  | 28 | 0.014 |  | -0.006 | 0.034 | 0.183 |  | 4 | 0.037 |  | -0.002 | 0.076 | 0.060 |  | 10 | 0.013 |  | -0.011 | 0.038 | 0.290 |
| Follow up period (month) | 26 | 0.006 |  | -0.055 | 0.068 | 0.840 |  | 29 | -0.006 |  | -0.048 | 0.036 | 0.776 |  | 4 | -0.045 |  | -0.351 | 0.261 | 0.774 |  | 12 | -0.002 |  | -0.037 | 0.033 | 0.916 |
| Treatment |  |  |  |  |  | 0.383 |  |  |  |  |  |  | 0.249 |  |  |  |  |  |  | 0.147 |  |  |  |  |  |  | 0.824 |
| Medication or system injection | 11 |  | -0.128 | -0.356 | 0.100 |  |  | 13 |  | -0.203 | -0.371 | -0.035 |  |  | 2 |  | -0.229 | -0.763 | 0.305 |  |  | 2 |  | 0.381 | -0.154 | 0.916 |  |
| Instillation | 10 |  | -0.234 | -0.483 | 0.016 |  |  | 10 |  | -0.087 | -0.279 | 0.110 |  |  | 1 |  | -0.539 | -1.007 | -0.071 |  |  | 6 |  | 0.251 | 0.015 | 0.486 |  |
| Intravesical injection | 3 |  | -0.629 | -1.146 | -0.113 |  |  | 4 |  | -0.483 | -0.888 | -0.078 |  |  |  |  |  |  |  |  |  | 3 |  | 0.251 | -0.160 | 0.662 |  |
| Others | 4 |  | -0.233 | -0.679 | 0.214 |  |  | 4 |  | -0.372 | -0.729 | -0.015 |  |  | 1 |  | 0.352 | -0.413 | 1.117 |  |  | 1 |  | -0.101 | -0.938 | 0.735 |  |
| Country |  |  |  |  |  | 0.449 |  |  |  |  |  |  | 0.307 |  |  |  |  |  |  | 0.078 |  |  |  |  |  |  | 0.757 |
| Western | 19 |  | -0.184 | -0.364 | -0.004 |  |  | 22 |  | -0.167 | -0.302 | -0.032 |  |  | 3 |  | -0.404 | -0.756 | -0.052 |  |  | 4 |  | 0.168 | -0.102 | 0.438 |  |
| Eastern | 9 |  | -0.312 | -0.592 | -0.032 |  |  | 9 |  | -0.307 | -0.538 | -0.075 |  |  | 1 |  | 0.352 | -0.413 | 1.117 |  |  | 7 |  | 0.294 | 0.052 | 0.536 |  |
| Middle East |  |  |  |  |  |  |  |  |  |  |  |  |  |  |  |  |  |  |  |  |  | 1 |  | 0.365 | -0.417 | 1.147 |  |
| Cystoscopy |  |  |  |  |  | 0.586 |  |  |  |  |  |  | 0.311 |  |  |  |  |  |  | 0.743 |  |  |  |  |  |  | 0.991 |
| Yes | 18 |  | -0.193 | -0.377 | -0.010 |  |  | 21 |  | -0.163 | -0.302 | -0.024 |  |  | 1 |  | -0.369 | -1.407 | 0.669 |  |  | 9 |  | 0.247 | 0.051 | 0.442 |  |
| No | 10 |  | -0.285 | -0.558 | -0.011 |  |  | 10 |  | -0.295 | -0.510 | -0.081 |  |  | 3 |  | -0.175 | -0.690 | 0.341 |  |  | 3 |  | 0.244 | -0.196 | 0.684 |  |
| Hunner lesion |  |  |  |  |  | 0.963 |  |  |  |  |  |  | 0.856 |  |  |  |  |  |  | 0.743 |  |  |  |  |  |  | 0.823 |
| HL+/HL- | 8 |  | -0.238 | -0.506 | 0.030 |  |  | 11 |  | -0.241 | -0.439 | -0.043 |  |  | 1 |  | -0.369 | -1.407 | 0.669 |  |  | 3 |  | 0.255 | -0.075 | 0.584 |  |
| HL- | 3 |  | -0.161 | -0.650 | 0.328 |  |  | 3 |  | -0.119 | -0.540 | 0.303 |  |  |  |  |  |  |  |  |  | 3 |  | 0.153 | -0.225 | 0.530 |  |
| - | 17 |  | -0.227 | -0.438 | -0.015 |  |  | 17 |  | -0.19 | -0.36 | -0.02 |  |  | 3 |  | -0.175 | -0.690 | 0.341 |  |  | 6 |  | 0.304 | 0.013 | 0.595 |  |
| PUF, pelvic pain, urgency, frequency symptom scale; FBV, functional bladder volume. *k*, number of effect sizes; β, regression coefficient; SMD, standardized mean difference; *p*-value from meta-regression analysis using the restricted maximum likelihood. | | | | | | | | | | | | | | | | | | | | | | | | | | | |

**eTable 6. Specific meta-analysis methodologies.**

| **Bayesian NMA** |
| --- |
| For Bayesian NMA, specific graphical analysis was completed using the “gemtc” package in R software v.4.2.1 (R Foundation for Statistical Computing). To compare the forty-seven included IC specific treatments, the prior distribution and likelihood were fed into a Markov chain Monte Carlo (MCMC) simulation, and the distribution with the best convergence of the posterior distribution was chosen. The MCMC simulation was used to determine the probability of a stable distribution and the area under the posterior distribution function. Finally, the posterior distribution was used to perform statistical reasoning for the treatment effect. Node-splitting assessments were performed to determine the association between the direct and indirect evidence for the consistency test. The surface under the cumulative ranking curve (SUCRA) was used to calculate the probability of each index test being the most effective treatment method based on a Bayesian approach using probability values to facilitate the interpretation of treatment performance; the larger the SUCRA value, the higher the rank of the intervention. |
| **Assessment of potential publication bias** |
| The funnel plot explains the publication bias using standard error as the measure of study size and SMD measures of treatment effect. In the absence of publication bias, the studies will be distributed symmetrically about the combined effect size. In addition, the study conducted the Egger linear regression method test for publication biases. |
| **Quality assessment** |
| The Cochrane Collaboration risk-of-bias (RoB) 2.0 tool was used to assess the risk of bias and methodological quality. The study evaluated five parameters: 1) the randomization process; 2) deviations from the intended interventions; 3) missing outcome data; 4) outcome measurements; and 5) selection of the reported results. Each domain was assigned a risk of bias rating of high, low, or unclear. The overall risk of bias was considered to be low if all domains were rated as "Low," some concerns if even one domain was rated as "Some concerns," and high risk if even one domain was rated as "High," or more than two domains were received the "Some concerns" rating. |
| **Data extraction and calculation** |
| Details regarding basic study information (first author, publication year, country and follow-up period), patient characteristics (number of patients and age) and technical aspects (inclusion and exclusion criteria and treatments) were extracted from the included articles using a predefined data extraction form. If a study included multiple treatment periods, the effect size at the end was calculated. If there were multiple dose ranges, the highest dose was set and the dose was converted based on the daily dose. Only studies that provided complete information were included in the final meta-analysis. For studies that did not report standard deviations, the combined standard deviation of the two groups was estimated. For studies that only reported medians, the mean and variance from the median, range and sample size were estimated. |
| **Study selection** |
| Duplicated publications were excluded, as were publications that did not contain original data, such as review articles, case reports, conference abstracts, editorials, letters and guidelines. Studies without a comparison group were excluded. Two investigators (SR Shim and JH Kim) independently analyzed the titles and abstracts for inclusion and exclusion criteria and examined the full-text articles using the same criteria. Then, the same authors independently extracted data using a data extraction form and the final inclusion articles were determined by all investigators following an evaluation discussion. References and data for each included study were carefully cross-checked to ensure that no overlapping data was present and to maintain the integrity of the meta-analysis. |

**eTable 7. PRISMA NMA Checklist of Items to Include When Reporting A Systematic Review Involving a Network Meta-analysis**

| **Section/Topic** | **Item #** | **Checklist Item** | **Reported on Page #** |
| --- | --- | --- | --- |
| **TITLE** |  |  |  |
| Title | 1 | Identify the report as a systematic review *incorporating a network meta-analysis (or related form of meta-analysis).* | ***1*** |
|  |  |  |  |
| **ABSTRACT** |  |  | ***2*** |
| Structured summary | 2 | Provide a structured summary including, as applicable:  **Background:** main objectives  **Methods:** data sources; study eligibility criteria, participants, and interventions; study appraisal; and *synthesis methods, such as network meta-analysis.*  **Results:** number of studies and participants identified; summary estimates with corresponding confidence/credible intervals; *treatment rankings may also be discussed. Authors may choose to summarize pairwise comparisons against a chosen treatment included in their analyses for brevity.*  **Discussion/Conclusions:** limitations; conclusions and implications of findings.  **Other:** primary source of funding; systematic review registration number with registry name. |  |
|  |  |  |  |
| **INTRODUCTION** |  |  |  |
| Rationale | 3 | Describe the rationale for the review in the context of what is already known*, including mention of why a network meta-analysis has been conducted.* | ***4*** |
| Objectives | 4 | Provide an explicit statement of questions being addressed, with reference to participants, interventions, comparisons, outcomes, and study design (PICOS). | 4-5 |
|  |  |  |  |
| **METHODS** |  |  | 5-8 |
| Protocol and registration | 5 | Indicate whether a review protocol exists and if and where it can be accessed (e.g., Web address); and, if available, provide registration information, including registration number. | 5 |
| Eligibility criteria | 6 | Specify study characteristics (e.g., PICOS, length of follow-up) and report characteristics (e.g., years considered, language, publication status) used as criteria for eligibility, giving rationale. *Clearly describe eligible treatments included in the treatment network, and note whether any have been clustered or merged into the same node (with justification).* | ***5-6*** |
| Information sources | 7 | Describe all information sources (e.g., databases with dates of coverage, contact with study authors to identify additional studies) in the search and date last searched. | 5 |
| Search | 8 | Present full electronic search strategy for at least one database, including any limits used, such that it could be repeated. | 5 |
| Study selection | 9 | State the process for selecting studies (i.e., screening, eligibility, included in systematic review, and, if applicable, included in the meta-analysis). | 6 & eTable 6 |
| Data collection process | 10 | Describe method of data extraction from reports (e.g., piloted forms, independently, in duplicate) and any processes for obtaining and confirming data from investigators. | 6 & eTable 6 |
| Data items | 11 | List and define all variables for which data were sought (e.g., PICOS, funding sources) and any assumptions and simplifications made. | 6 & eTable 6 |
| **Geometry of the network** | **S1** | Describe methods used to explore the geometry of the treatment network under study and potential biases related to it. This should include how the evidence base has been graphically summarized for presentation, and what characteristics were compiled and used to describe the evidence base to readers. | 6 & eTable 6 |
| Risk of bias within individual studies | 12 | Describe methods used for assessing risk of bias of individual studies (including specification of whether this was done at the study or outcome level), and how this information is to be used in any data synthesis. | 6 & eTable 6 |
| Summary measures | 13 | State the principal summary measures (e.g., risk ratio, difference in means). *Also describe the use of additional summary measures assessed, such as treatment rankings and surface under the cumulative ranking curve (SUCRA) values, as well as modified approaches used to present summary findings from meta-analyses.* | 6 & eTable 6 |
| Planned methods of analysis | 14 | Describe the methods of handling data and combining results of studies for each network meta-analysis. This should include, but not be limited to:   - *Handling of multi-arm trials;* - *Selection of variance structure;* - *Selection of prior distributions in Bayesian analyses; and* - *Assessment of model fit.* | 6 |
| **Assessment of Inconsistency** | **S2** | Describe the statistical methods used to evaluate the agreement of direct and indirect evidence in the treatment network(s) studied. Describe efforts taken to address its presence when found. | 6 & eTable 6 |
| Risk of bias across studies | 15 | Specify any assessment of risk of bias that may affect the cumulative evidence (e.g., publication bias, selective reporting within studies). | 6 & eTable 6 |
| Additional analyses | 16 | Describe methods of additional analyses if done, indicating which were pre-specified. This may include, but not be limited to, the following:   - Sensitivity or subgroup analyses; - Meta-regression analyses; - *Alternative formulations of the treatment network; and* - *Use of alternative prior distributions for Bayesian analyses (if applicable).* | 6 & eTable 6 |
|  |  |  |  |
| **RESULTS†** |  |  | ***7-9*** |
| Study selection | 17 | Give numbers of studies screened, assessed for eligibility, and included in the review, with reasons for exclusions at each stage, ideally with a flow diagram. | 7 |
| **Presentation of network structure** | **S3** | Provide a network graph of the included studies to enable visualization of the geometry of the treatment network. | ***eFigures*** |
| **Summary of network geometry** | **S4** | Provide a brief overview of characteristics of the treatment network. This may include commentary on the abundance of trials and randomized patients for the different interventions and pairwise comparisons in the network, gaps of evidence in the treatment network, and potential biases reflected by the network structure. | ***8 & eFigures*** |
| Study characteristics | 18 | For each study, present characteristics for which data were extracted (e.g., study size, PICOS, follow-up period) and provide the citations. | 7, eTables |
| Risk of bias within studies | 19 | Present data on risk of bias of each study and, if available, any outcome level assessment. | 9 & ***eFigures*** |
| Results of individual studies | 20 | For all outcomes considered (benefits or harms), present, for each study: 1) simple summary data for each intervention group, and 2) effect estimates and confidence intervals. *Modified approaches may be needed to deal with information from larger networks.* | ***7-8, Figures*** |
| Synthesis of results | 21 | Present results of each meta-analysis done, including confidence/credible intervals. *In larger networks, authors may focus on comparisons versus a particular comparator (e.g. placebo or standard care), with full findings presented in an appendix. League tables and forest plots may be considered to summarize pairwise comparisons.* If additional summary measures were explored (such as treatment rankings), these should also be presented. | ***7-8, Figures*** |
| **Exploration for inconsistency** | **S5** | Describe results from investigations of inconsistency. This may include such information as measures of model fit to compare consistency and inconsistency models, *P* values from statistical tests, or summary of inconsistency estimates from different parts of the treatment network. | ***9*** |
| Risk of bias across studies | 22 | Present results of any assessment of risk of bias across studies for the evidence base being studied. | 9 & ***eFigure*** |
| Results of additional analyses | 23 | Give results of additional analyses, if done (e.g., sensitivity or subgroup analyses, meta-regression analyses*, alternative network geometries studied, alternative choice of prior distributions for Bayesian analyses,* and so forth). | 9 & ***eFigures*** |
|  |  |  |  |
| **DISCUSSION** |  |  |  |
| Summary of evidence | 24 | Summarize the main findings, including the strength of evidence for each main outcome; consider their relevance to key groups (e.g., healthcare providers, users, and policy-makers). | 10, 14 |
| Limitations | 25 | Discuss limitations at study and outcome level (e.g., risk of bias), and at review level (e.g., incomplete retrieval of identified research, reporting bias). *Comment on the validity of the assumptions, such as transitivity and consistency. Comment on any concerns regarding network geometry (e.g., avoidance of certain comparisons).* | 13 |
| Conclusions | 26 | Provide a general interpretation of the results in the context of other evidence, and implications for future research. | 14 |
|  |  |  |  |
| **FUNDING** |  |  |  |
| Funding | 27 | Describe sources of funding for the systematic review and other support (e.g., supply of data); role of funders for the systematic review. This should also include information regarding whether funding has been received from manufacturers of treatments in the network and/or whether some of the authors are content experts with professional conflicts of interest that could affect use of treatments in the network. | ***15*** |

PICOS = population, intervention, comparators, outcomes, study design.

* Text in italics indicateS wording specific to reporting of network meta-analyses that has been added to guidance from the PRISMA statement.

† Authors may wish to plan for use of appendices to present all relevant information in full detail for items in this section.
